# Oxidative Functionalization of Woody Biochar for Hexavalent Chromium Detoxification: Adsorption-Coupled Reduction and Dual-Phase Remediation

**DOI:** 10.3390/molecules31132384

**Published:** 2026-07-06

**Authors:** Sitong Li, Junfeng Tang, Zihan Su, Lipin Ren, Yonglong Wu, Guiji Guo, Jinghao Rao, Meiqin Zhou, Yue Fan

**Affiliations:** 1 College of Civil Engineering, Sichuan Agricultural University, Dujiangyan 611830, China; 19981517227@163.com (S.L.); suzihan200603@163.com (Z.S.); 13980240859@163.com (L.R.); 18884601651@163.com (Y.W.); ggj846075901@163.com (G.G.); 19113206087@163.com (J.R.); 18808241887@163.com (M.Z.); 19583588985@163.com (Y.F.); 2 Sichuan Higher Education Engineering Research Center for Disaster Prevention and Mitigation of Village Construction, Sichuan Agricultural University, Dujiangyan 611830, China; 3 Engineering Research Center of Catastrophic Prophylaxis and Treatment of Road & Traffic Safety of Ministry of Education, Changsha University of Science & Technology, Changsha 410114, China

**Keywords:** oxidative functionalization, hexavalent chromium, functionalized biochar, adsorption-coupled reduction, environmental remediation

## Abstract

To address the ecological risks associated with highly mobile hexavalent chromium [Cr(VI)], woody biochar was functionalized with hydrogen peroxide (H_2_O_2_) to develop a dual-phase remediation material (H-BC) for aqueous and soil environments. Batch post-contact isotherm fitting yielded a Langmuir-fitted/extrapolated apparent retention capacity q_m_ of 77.44 mg/g at 328 K. This value reflects enhanced overall Cr(VI)-derived retention within the tested concentration range, rather than increased electrostatic affinity for chromate oxyanions. Empirical kinetic diagnostics and FTIR/XPS results were consistent with adsorption-coupled interfacial reduction, while DFT analysis provided qualitative support for the enhanced electronic responsiveness of H-BC. The OFG-enriched interface may facilitate short-range, non-electrostatic interfacial interactions and stabilize surface-associated Cr(III). Temperature-dependent apparent isotherm fitting suggested that elevated temperature favored the overall Cr(VI)-derived retention process under the tested conditions, and should not be interpreted as rigorous standard-state adsorption thermodynamics. Continuous-flow column leaching and accelerated wet–dry (W–D) aging experiments demonstrated that H-BC substantially suppressed the mobility of operationally filtered Cr(VI), achieving a maximum filtered-Cr(VI)-based retention efficiency of 99.98% under cyclic drying–rewetting conditions. Spatial configuration analysis indicated that homogeneous incorporation of H-BC improved soil–biochar contact and was more effective than stratified placement in limiting vertical filtered-Cr(VI) migration. Overall, oxidatively functionalized H-BC shows promise as a biomass-derived amendment for reducing Cr(VI) mobility in complex environmental matrices, although complete chromium mass redistribution will require future total-Cr and Cr(III)-resolved analyses.

## 1. Introduction

The continuous release of industrial effluents has led to significant accumulation of hexavalent chromium [Cr(VI)] in terrestrial environments, presenting critical ecological and public health concerns [1]. Given its high mobility, mutagenicity, and carcinogenicity, Cr(VI) easily penetrates groundwater systems, constituting a major challenge in environmental remediation. Within environmental matrices, Cr(VI) predominantly occurs as highly soluble oxyanions (such as CrO_4_^2−^, HCrO_4_^−^, and Cr_2_O_7_^2−^) with remarkable transport ability across both fluid and solid interfaces. The intrinsic resistance of these anions to natural sequestration promotes their rapid downward movement through advective-dispersive pathways, heavily endangering underlying aquifers [2]. This immobilization bottleneck becomes especially acute in semi-arid, nutrient-poor areas. In this study, the sandy loam collected from Songpan County (Sichuan, China) was used as a native, non-industrially contaminated pedological matrix, in which chromium is considered to be associated with natural/geogenic background rather than known industrial inputs. This soil exhibits a severe deficiency in soil organic matter (SOM ≈ 0.5%), which limits its natural reductive buffering capacity and availability of electron donors. Therefore, after controlled dichromate amendment, this low-SOM sandy loam provides a representative model system for evaluating Cr(VI) mobility and leaching under weak natural attenuation conditions [3].

To overcome these site-specific challenges, biochar has gained attention as a structurally adaptable and economically viable carbon platform for heavy metal stabilization [3]. While porosity and surface area provide a physical basis for solute retention, pristine biochar often shows limited Cr(VI) removal at circumneutral pH as its surface is negatively charged and its density of chemically reactive sites is low [4,5]. H_2_O_2_ oxidation increases the abundance of carboxyl, hydroxyl, carbonyl, and ether-type groups, thereby increasing surface polarity and chemical heterogeneity [4]. However, dissociation of acidic groups also increases the negative surface charge; therefore, improved Cr(VI)-derived retention cannot be attributed to stronger Coulombic attraction toward chromate oxyanions. Instead, oxygenated surface domains may support short-range non-electrostatic association, facilitate reaction-coupled electron transfer at residual redox-active sites, and coordinate the resulting Cr(III) species [1,3,6,7].

Guided by these mechanistic insights, H_2_O_2_-functionalized woody biochar (H-BC) was developed as a dual-phase Cr(VI) remediation material for both aqueous and soil environments. Building on established oxidative-functionalization principles, this study integrates aqueous-phase adsorption and interfacial reduction analysis with soil-phase immobilization assessment, thereby extending the evaluation of H-BC beyond conventional batch adsorption [3,6,7]. To verify its practical utility, a comprehensive framework was designed to elucidate micro-interfacial physicochemical dynamics and assess the macroscopic mitigation of Cr(VI) mobility under environmental stresses, including accelerated wet–dry (W-D) aging. By combining kinetic and isotherm modeling with spectroscopic techniques, including FTIR and XPS, and supplementing with DFT-based electronic-structure analysis, this study systematically investigates the adsorption-coupled reduction pathway of Cr(VI) on H-BC. Continuous-flow column leaching, W-D aging simulations, and spatial amendment-placement experiments were further employed to evaluate the environmental immobilization stability of H-BC under hydrodynamic flushing and cyclic drying–rewetting conditions. Ultimately, this research positions surface-engineered woody biochar as a scalable and technically feasible amendment for long-term Cr(VI) remediation, particularly in low-organic-matter soils where natural reductive buffering and contaminant retention are limited [2].

## 2. Results and Discussion

### 2.1. Physicochemical Properties of Biochar

#### 2.1.1. Morphology and Elemental Composition of Biochar

**Figure 1:**
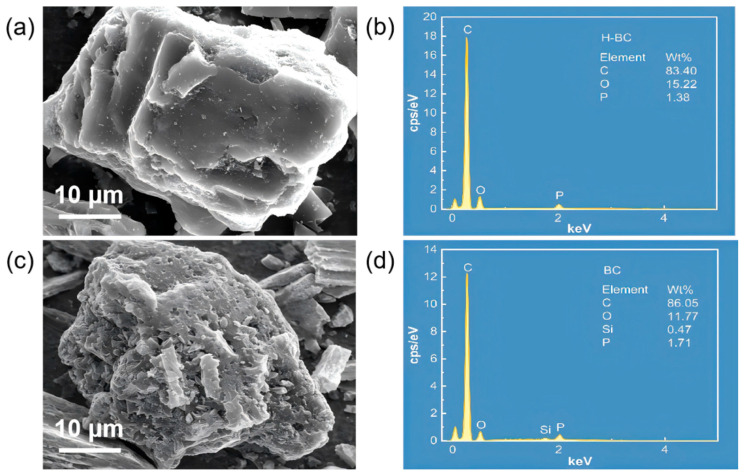
SEM images and EDS spectra of pristine biochar (BC) and H_2_O_2_-modified biochar (H-BC). (**a**) SEM image of BC; (**b**) corresponding EDS spectrum of BC; (**c**) SEM image of H-BC; (**d**) corresponding EDS spectrum of H-BC. The H_2_O_2_-modified biochar shows a rougher surface compared with pristine BC. EDS results indicate an increase in oxygen content after modification.

SEM–EDS analysis was used to examine the morphological and elemental changes induced by H_2_O_2_ functionalization. As shown in Figure 1a, pristine biochar (BC) exhibited a relatively compact surface with limited visible pore openings, which may restrict external access to internal adsorption sites. After H_2_O_2_ treatment, H-BC displayed a rougher and more fragmented surface with newly exposed pore entrances and surface defects (Figure 1c). These morphological changes are consistent with oxidative etching of the carbon matrix and can improve the accessibility of reactive surface domains [8,9,10].

Quantitative EDS microanalysis corroborated concurrent chemical functionalization (Figure 1b,d). Following H_2_O_2_ treatment, the oxygen content increased from 11.77% to 15.22%, while the carbon content decreased from 86.05% to 83.40%. The increased O/C mass ratio indicates the formation of a more polar and chemically heterogeneous interface. However, as H-BC remains negatively charged under circumneutral conditions, this change should not be interpreted as enhanced electrostatic attraction toward anionic Cr(VI) species [4,8].

Structurally, the roughened surface and newly exposed pore entrances observed by SEM suggest local surface opening and greater exposure of selected reactive domains. However, these local morphological features should not be interpreted as an increase in the overall N_2_-accessible surface area or total pore volume, as confirmed by the BET results [8,11]. Chemically, the increased oxygen content indicates enrichment of oxygen-containing functional groups (OFGs), which can enhance surface polarity and provide chemically heterogeneous domains that participate in short-range interfacial interactions, redox transformation, and subsequent Cr(III) stabilization [3,5]. Overall, SEM–EDS indicates that H_2_O_2_ modification reshapes H-BC mainly through local oxidative etching and surface functionalization, whereas the quantitative pore-structure changes are evaluated separately by BET analysis [5,12].

#### 2.1.2. Surface Functional Group Characterization of Biochar via FTIR

**Figure 2:**
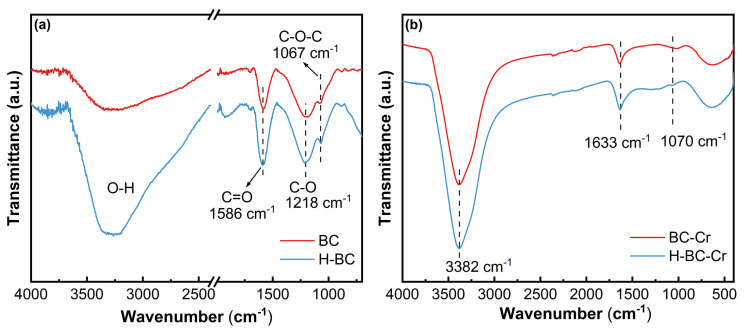
FTIR spectra of biochars before and after Cr(VI) adsorption. (**a**) Pristine biochar (BC) and H_2_O_2_-modified biochar (H-BC); (**b**) BC and H-BC after adsorption. The spectra show typical bands corresponding to O–H, C=O/aromatic C=C, and C–O/C–O–C groups. H_2_O_2_ modification increases the intensity of oxygen-containing functional groups, while changes after adsorption suggest their participation in Cr(VI) uptake.

FTIR spectroscopy was used to examine changes in surface functional groups of the biochar matrices. As illustrated in the pre-adsorption spectra (Figure 2a), both BC and H-BC displayed bands at approximately 3400, 1586, 1218, and 1067 cm^−1^. The broad feature near 3400 cm^−1^ is assigned to surface hydroxyl groups, with possible contribution from strongly bound/adsorbed water [13]. The band near 1586 cm^−1^ is attributed to overlapping carbonyl and aromatic skeletal vibrations, whereas the bands at 1218 and 1067 cm^−1^ correspond to C–O/C–O–C moieties [3,4,13]. The increased intensity of oxygen-related bands after H_2_O_2_ treatment is consistent with OFG enrichment and the formation of a more polar and chemically heterogeneous interface [3,5,8,14].

After Cr(VI) adsorption (Figure 2b), the FTIR spectra showed clear changes, with H-BC exhibiting more pronounced variations than BC. The broad –OH band near 3400 cm^−1^ decreased in intensity and shifted to 3382 cm^−1^, while the carbonyl/aromatic skeletal vibration shifted from 1586 to 1633 cm^−1^. Changes were also observed in the C–O/C–O–C region, with a band appearing near 1070 cm^−1^. These peak shifts and intensity variations support the participation of oxygen-containing groups in the interfacial retention of chromium species [1,15].

When considered together with the O 1s and Cr 2p XPS results and the electrokinetic characteristics of H-BC [4,14], the FTIR changes are consistent with chemically specific, non-electrostatic interfacial interactions, adsorption-coupled reduction, and stabilization of surface-associated Cr(III). The more pronounced spectral response of H-BC further indicates a greater contribution of its oxygenated surface domains to the overall Cr(VI)-derived retention process [5].

#### 2.1.3. XPS Characterization of Chromium Species on Biochar Surfaces

**Figure 3:**
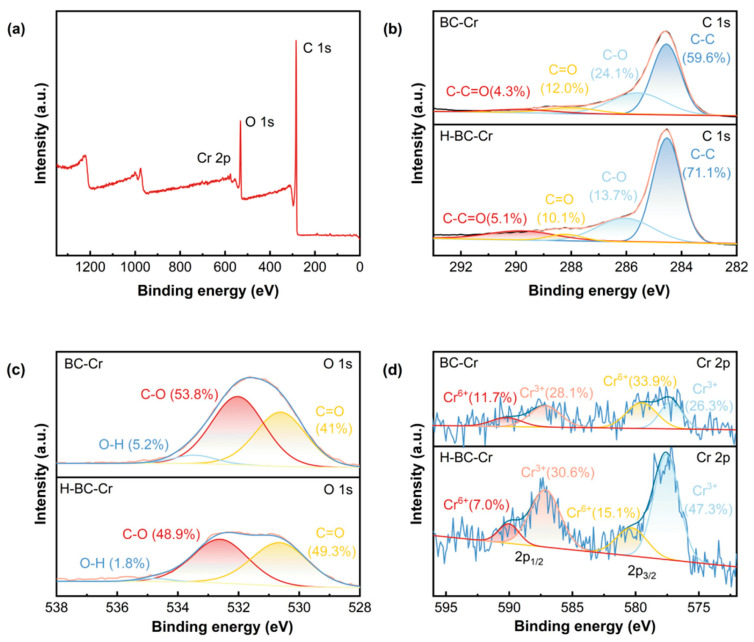
XPS spectra of Cr-loaded pristine biochar (BC-Cr) and H_2_O_2_-modified biochar (H-BC-Cr): (**a**) survey spectra; (**b**) high-resolution C 1s spectra; (**c**) high-resolution O 1s spectra; and (**d**) high-resolution Cr 2p spectra showing Cr(III) and Cr(VI) species. The spectral changes indicate surface functionalization and partial Cr(VI) reduction after adsorption.

To delineate the interfacial redox mechanisms and the definitive speciation of the sequestered chromium, X-ray photoelectron spectroscopy (XPS) was executed on the post-adsorption matrices. The wide-scan survey spectra (Figure 3a) confirmed the presence of Cr together with the intrinsic C and O signals, indicating successful Cr immobilization on the biochar surfaces. Deconvolution of the high-resolution C 1s spectra (Figure 3b), calibrated against the carbon-framework C–C peak at 284.8 eV, revealed characteristic contributions from C–O, C=O, and ester/carboxyl carbonyl (O–C=O) functionalities [16]. The C–C component at 284.8 eV corresponds to the residual biochar carbon framework and serves as the binding-energy reference; thus, its relative variation should be regarded as a normalized change within the C 1s surface composition rather than as evidence of newly formed C–C bonds. The slight increase in the O–C=O contribution from 4.3% in BC-Cr to 5.1% in H-BC-Cr indicates the retention and modest enrichment of oxygenated binding sites after Cr(VI) remediation [3]. This result indicates that oxygenated binding sites remained detectable after Cr(VI) adsorption, supporting their involvement in chromium immobilization [3,4,5].

Further mechanistic insight is provided by the O 1s spectra (Figure 3c), which were deconvoluted into C=O, C–O, and O–H components. The C=O proportion increased from 41.0% in BC-Cr to 49.3% in H-BC-Cr, accompanied by decreases in C–O (53.8% to 48.9%) and O–H (5.2% to 1.8%). This trend does not indicate suppression of Cr(VI) reduction; rather, it is consistent with the partial oxidation of labile C–O/O–H groups during interfacial electron transfer to Cr(VI). In this process, C–O/O–H moieties may serve as electron-donating sites, whereas carbonyl/carboxyl groups enhance surface polarity, coordination affinity, and stabilization of reduced Cr(III) species [1,3,5,17,18]. This observation helps clarify the apparently counterintuitive role of oxidative functionalization. H_2_O_2_ treatment does not make H-BC a stronger bulk reductant; instead, it generates a mixed oxygenated interface where chromate species are more effectively adsorbed and positioned near residual redox-active C–O/O–H groups and the carbon framework. Thus, enhanced Cr(VI) reduction on H-BC is best interpreted as an adsorption-localized interfacial process rather than a simple increase in the intrinsic reducing power of the oxidized biochar [3,6,7].

This interpretation is further supported by the Cr 2p core-level spectra (Figure 3d), which provide direct spectroscopic evidence for chromium redox transformation through the characteristic spin–orbit split doublets (Cr 2p1/2 and Cr 2p3/2). Deconvolution of the Cr 2p region showed that the surface-bound Cr(III) fraction increased to 77.9% on H-BC-Cr, compared with 54.4% on BC-Cr. The higher abundance of Cr(III) on H-BC-Cr is consistent with an adsorption-coupled reduction pathway, in which Cr(VI) species are first captured by oxygenated surface sites and subsequently undergo partial interfacial electron transfer to form more stable Cr(III) species [5,15,19,20].

However, the Cr 2p result should be interpreted as surface-sensitive evidence of chromium speciation on the recovered biochar surface rather than as a complete Cr mass balance. Thus, the higher Cr(III) fraction on H-BC-Cr supports adsorption-coupled interfacial reduction, but this result alone cannot distinguish the relative contributions of aqueous Cr(VI) adsorption, potential dissolved Cr(III) formation, colloid-associated chromium retention, and solid-phase chromium accumulation.

#### 2.1.4. Zeta Potential and pH_pzc_ of Pristine and Modified Biochar

**Figure 4:**
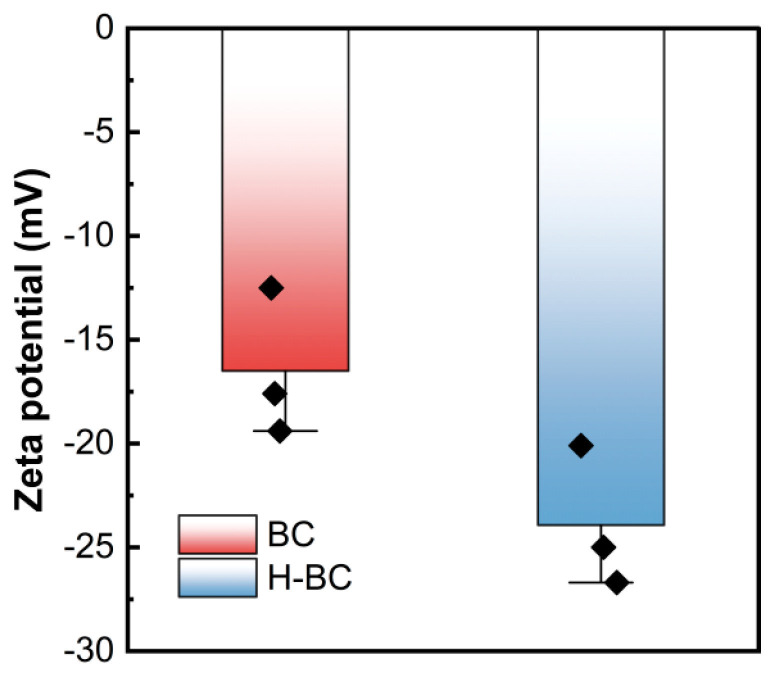
Zeta potential and pH_PZC_ of pristine biochar (BC) and H_2_O_2_-modified biochar (H-BC). In the figure, BC is shown in red and H-BC is shown in blue. Zeta potentials were measured in deionized water suspensions at pH 6.5 and 25 °C. H_2_O_2_ modification shifted the apparent zeta potential from −16.5 ± 3.6 to −23.9 ± 3.4 mV and decreased the pH_PZC_ from 4.0–4.5 to 2.0–2.5.

Electrokinetic analyses, including zeta potential and point of zero charge (pH_PZC_) determinations, were conducted to evaluate the surface charge evolution following oxidative functionalization. As zeta potential depends strongly on the measurement medium, especially pH and ionic strength, the values reported here are interpreted under the defined aqueous suspension conditions described in Section 3.3 rather than as solution-independent surface constants [21,22,23]. As shown in Figure 4, BC exhibited an apparent zeta potential of −16.5 ± 3.6 mV at pH 6.5 and 25 °C, whereas H-BC showed a more negative apparent zeta potential of −23.9 ± 3.4 mV under the same conditions. The corresponding conductivities were 0.024 ± 0.002 and 0.039 ± 0.003 mS/cm for BC and H-BC, respectively. Concurrently, the pH_PZC_ decreased from 4.0–4.5 for BC to 2.0–2.5 for H-BC, suggesting stronger acidic surface character after oxidative treatment. These electrokinetic changes are consistent with the enrichment of ionizable oxygen-containing functional groups, including carboxyl- and phenolic-type moieties [4,14]. Importantly, the zeta potential and pH_PZC_ results are interpreted here as comparative indicators of surface charge evolution and should be considered together with the FTIR and XPS evidence.

The initial batch adsorption pH (6.0 ± 0.1) and the ζ-potential measurement pH (6.5) were both above the pH_pzc_ of H-BC (2.0–2.5), consistent with a net negatively charged surface under circumneutral conditions. These electrokinetic characteristics indicate that simple Coulombic attraction is not the principal pathway governing chromate retention by H-BC. Instead, the higher overall Cr(VI)-derived retention is consistent with chemically specific, non-electrostatic interfacial interactions and adsorption-coupled reduction at heterogeneous oxygenated surface domains. Residual C–O/O–H moieties may participate in interfacial electron transfer, while carbonyl- and carboxylate-type groups may contribute to the coordination and stabilization of surface-associated Cr(III) [1,5,17]. Considered together with the FTIR and XPS results, the electrokinetic data support a reaction-coupled interfacial retention mechanism rather than a purely electrostatic adsorption process [1,17].

#### 2.1.5. BET Surface Area and Pore-Structure Analysis

**Figure 5:**
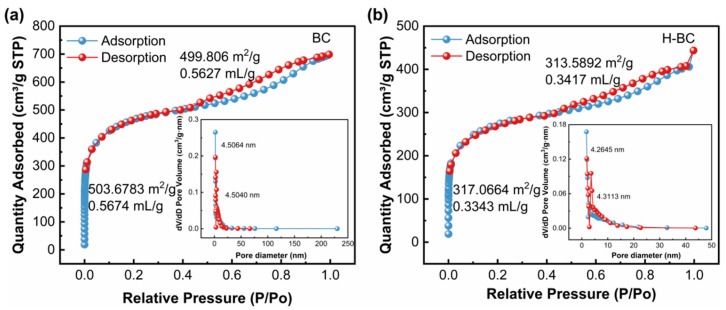
N_2_ adsorption–desorption isotherms and pore-size distribution of pristine biochar (BC) and H_2_O_2_-modified biochar (H-BC): (**a**) BC and (**b**) H-BC. BET analysis shows that H_2_O_2_ modification decreased the specific surface area and total pore volume, while the dominant pore-size peak remained at approximately 4.3–4.5 nm, indicating retention, rather than enhancement, of the mesoporous framework after oxidative functionalization.

To further quantify the textural differences between pristine biochar and H_2_O_2_-modified biochar, N_2_ adsorption–desorption analysis was performed to determine the BET specific surface area, total pore volume, and pore-size distribution of BC and H-BC. As summarized in Table 1 and Figure 5, BC exhibited a specific surface area of 503.7 m^2^/g and a total pore volume of 0.5674 mL/g. After H_2_O_2_ modification, the BET surface area decreased to 317.1 m^2^/g, and the total pore volume decreased to 0.3343 mL/g. This result demonstrates that H_2_O_2_ functionalization decreased the overall N_2_-accessible textural porosity of the biochar. The decrease may be associated with pore-wall oxidation, partial occupation or narrowing of micropores by newly introduced oxygen-containing functional groups, and limited collapse of unstable pore domains during wet oxidation [3,5]. Therefore, H-BC should not be described as having increased total porosity after modification.

Despite the decrease in BET surface area and total pore volume, the dominant pore-size peak remained at approximately 4.3–4.5 nm, indicating that a mesoporous transport framework was still retained. This retained mesoporosity may facilitate solute access to reactive surface regions, but it does not imply an increase in total pore volume. Accordingly, the enhanced Cr(VI)-derived retention by H-BC should not be attributed to surface-area expansion or physical pore filling. Rather, it is more reasonably associated with the OFG-enriched interface, improved local exposure of selected surface domains (observed by SEM), and retained mesoporous transport pathways [3,5,20,24].

Taken together, SEM and BET provide complementary but non-equivalent information. SEM–EDS shows local surface roughening, fragmentation, and increased oxygen content (from 11.77% to 15.22%), whereas BET confirms a decrease in internal N_2_-accessible surface area and total pore volume. Thus, H_2_O_2_ treatment is best interpreted as creating an OFG-enriched, locally etched mesoporous interface rather than increasing the overall porosity of the biochar [20,24].

### 2.2. Cr(VI) Adsorption Kinetics on Modified Biochar

**Figure 6:**
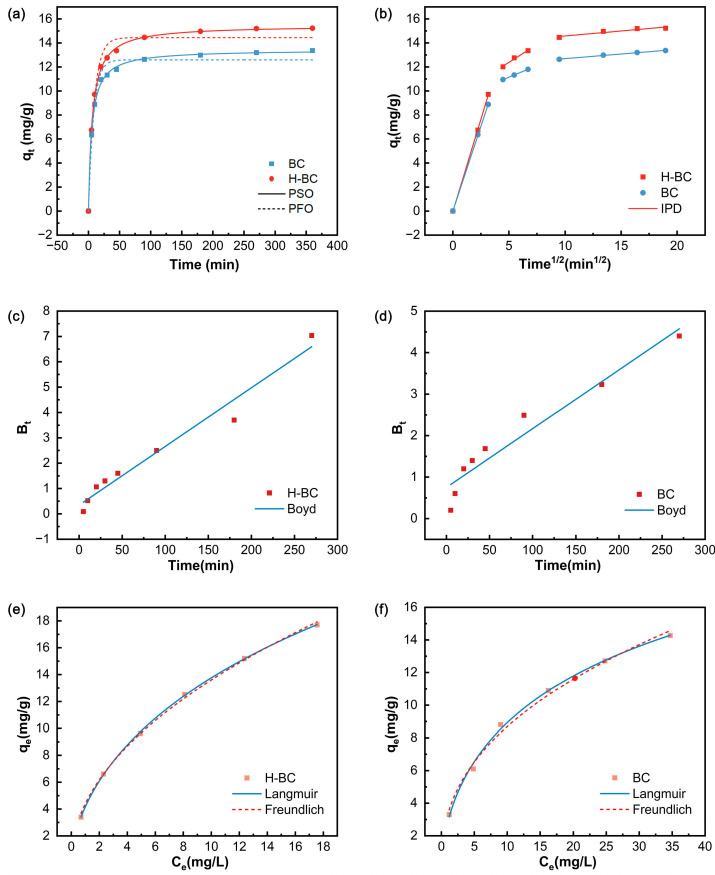
Kinetic, diffusion, and apparent isotherm fitting analyses for Cr(VI)-derived retention by BC and H-BC. (**a**) Pseudo-first-order (PFO) and pseudo-second-order (PSO) kinetic fittings for Cr(VI) uptake by BC and H-BC; (**b**) Weber–Morris intra-particle diffusion (IPD) fitting plots; (**c**) Boyd-model fitting plot for H-BC; (**d**) Boyd-model fitting plot for BC; (**e**) Langmuir and Freundlich apparent isotherm fittings for H-BC; and (**f**) Langmuir and Freundlich apparent isotherm fittings for BC. The Boyd plots were used to further evaluate the relative contributions of external film diffusion and intra-particle diffusion. The non-zero intercepts of the Boyd fitted lines indicate that intra-particle diffusion was not the sole rate-controlling step and that film diffusion also contributed to the overall Cr(VI) uptake process.

The temporal adsorption trajectories (Figure 6a) for both BC and H-BC exhibited a distinct biphasic nature. An initial rapid Cr(VI) uptake occurred within the first 45 min, driven by the abundance of highly accessible vacant sites, followed by a decelerated phase as these active centers approached saturation, ultimately attaining dynamic equilibrium between 180 and 270 min. Non-linear regression analysis showed that the pseudo-second-order (PSO) model provided a better empirical description of the kinetic data than the pseudo-first-order (PFO) model, as indicated by higher R^2^ values and lower RMSE, AIC, and BIC values (Table 2). However, PSO is an empirical kinetic model and does not by itself prove chemisorption or electron transfer. Therefore, the PSO fit is interpreted here as being consistent with surface-controlled uptake under the tested conditions, whereas the adsorption-coupled reduction mechanism is inferred from the combined kinetic, FTIR, XPS, electrokinetic, IPD, and DFT-related evidence [17,25,26].

To further decode the sequential mass transfer resistances, the Weber-Morris intra-particle diffusion (IPD) model was utilized (Figure 6b), and the corresponding fitted parameters are summarized in Table 3. The multilinear IPD plots delineate three progressive stages: instantaneous external boundary layer diffusion, subsequent intra-particle pore diffusion, and final equilibrium. The initial film diffusion rate (k_p1_) for H-BC (3.1070) surpassed that of BC (2.8382), reflecting reduced boundary layer resistance following oxidative modification. Similarly, the higher intra-particle diffusion rate (k_p2_) of H-BC (0.6666) compared with BC (0.3783) suggests improved internal transport and accessibility of Cr(VI) species to reactive sites. The progressive deceleration across the stages (k_p1_ > k_p2_ > k_p3_) denotes the gradual depletion of accessible binding sites. Moreover, the consistently non-zero intercepts (C_3_ > C_2_ > C_1_ > 0) confirm a mixed-control mass transfer regime, wherein the adsorption kinetics are synergistically governed by both external film diffusion and internal pore transport rather than a single kinetic bottleneck [1,27].

To further distinguish the relative contributions of film diffusion and intra-particle diffusion, the Boyd model was additionally applied to the adsorption kinetic data, and the corresponding fitted parameters are summarized in Table 4. The Boyd plots for BC (Figure 6d) and H-BC (Figure 6c) showed acceptable linearity, with R^2^ values of 0.93539 and 0.96676, respectively. However, the fitted lines did not extrapolate through the origin, as reflected by positive intercepts of 0.75077 for BC and 0.34730 for H-BC. According to the Boyd diffusion criterion, this deviation indicates that intra-particle diffusion was not the sole rate-controlling step and that external film diffusion also contributed to the overall Cr(VI) uptake process. In addition, the Boyd slope of H-BC (0.02313) was higher than that of BC (0.01416), suggesting faster diffusion-related transport after H_2_O_2_ functionalization. These results support the IPD-based interpretation that Cr(VI) adsorption proceeded through a mixed diffusion regime rather than exclusive intra-particle diffusion control.

Building on the mixed-control mass-transfer behavior jointly indicated by the IPD and Boyd models, the kinetic evaluation suggests that H_2_O_2_ functionalization improved Cr(VI) uptake through coupled interfacial chemical reactivity and diffusion-related transport effects. The PSO model provided the best empirical description of the time-course data for both BC and H-BC, but the PSO rate parameter should not be interpreted as direct proof of chemisorption or electron transfer. Rather, the role of OFGs is supported by the agreement among kinetic behavior, FTIR, XPS, electrokinetic, and DFT-related evidence. Concurrently, the IPD and Boyd parameters suggest improved access of Cr(VI) to reactive domains, which may be related to SEM-observed local surface etching, retained mesoporous pathways, and reduced diffusion resistance [6,8]. Therefore, the enhanced kinetic performance of H-BC is attributed to OFG-mediated surface reactivity and locally accessible transport pathways, rather than to PSO fitting or a single diffusion-control mechanism alone [4,5,8,11,17].

### 2.3. Apparent Isotherm Fitting of Cr(VI)-Derived Retention on Biochar and H_2_O_2_-Modified Biochar

To evaluate the concentration-dependent Cr(VI)-derived retention behavior, post-contact adsorption datasets were obtained at 298, 313, and 328 K and fitted with the Langmuir and Freundlich equations; the corresponding apparent isotherm parameters and temperature-dependent retention indicators are summarized in Table 5. Because Cr(VI) uptake on BC and H-BC involves adsorption-coupled partial reduction to Cr(III), these equations are used here as apparent isotherm fitting models rather than as proof of reversible thermodynamic equilibrium. The Langmuir equation provided higher goodness-of-fit values than the Freundlich equation over the tested concentration range (R^2^ > 0.993), indicating that it offered a useful empirical description of the experimental datasets [1,26,28]. However, as no clear saturation plateau was experimentally reached, the Langmuir-derived q_m_ should be interpreted as an extrapolated apparent retention capacity under the assumptions of the model, rather than as a directly observed maximum capacity [1,28]. Under the present V/m ratio (0.1 L/0.5 g), the maximum experimentally possible uptake at C_0_ = 106.06 mg/L is approximately 21.2 mg/g, even assuming complete Cr(VI) removal. In the isotherm datasets, the highest directly measured q_e_ was 17.70 mg/g for H-BC at 328 K, with an observed C_e_ range of 0.6933–17.5614 mg/L for this fitting condition and 0.6933–61.0078 mg/L across all tested isotherm datasets. Therefore, the fitted q_m_ values, including 77.44 mg/g for H-BC at 328 K, are used for comparative model-based evaluation and should not be interpreted as directly observed uptake capacities. Under this restricted interpretation, H-BC showed a Langmuir-derived apparent retention capacity of 77.44 mg/g at 328 K, approximately 2.8 times that of pristine BC (27.83 mg/g) under identical fitting conditions [4,5,17].

The fitted retention capacity increased with temperature, suggesting that elevated temperature favored Cr(VI)-derived retention under the present experimental conditions. This trend may be associated with enhanced diffusion, partial dehydration of Cr(VI) oxyanions, increased accessibility of reactive interfacial sites, and/or activation-energy-demanding reduction on specific OFG-rich surface domains [1,15,28]. As Cr(VI) retention is coupled with partial reduction to Cr(III), the observed temperature dependence should be interpreted as an apparent enhancement in the overall retention–reduction process rather than as direct evidence of endothermic adsorption alone. Taken together, the isotherm fits indicate greater overall Cr(VI)-derived retention by H-BC within the tested concentration range. Given the net negative surface charge of H-BC under the experimental conditions, this behavior is not attributed to increased electrostatic affinity for chromate oxyanions. Instead, it is consistent with the combined contributions of mass transfer through the retained mesoporous framework, short-range non-electrostatic interfacial interactions, partial Cr(VI) reduction, and stabilization of surface-associated Cr(III) at oxygenated surface domains [4,5,26].

Although the present results support an adsorption-coupled reduction pathway, the potential contribution of biochar-derived dissolved carbonaceous species should also be considered. As the batch aliquots were analyzed for residual Cr(VI) after 0.45-μm filtration and DPC colorimetry, dissolved TOC and aqueous Cr(III)/total Cr were not quantified in this analytical design. Thus, homogeneous Cr(VI) reduction mediated by leached carbonaceous species cannot be excluded. Future work should resolve this pathway by pairing TOC with filtered and unfiltered total Cr, dissolved Cr(III), and Cr(VI) measurements, thereby distinguishing adsorption, aqueous reduction, and particle-associated retention.

To place the Langmuir-derived apparent capacity of H-BC in context, the value obtained in this study was compared with selected biochar-based Cr(VI) adsorbents reported in the literature (Table 6). As adsorption capacity is strongly affected by initial Cr(VI) concentration, adsorbent dosage, pH, contact time, temperature, and solution chemistry, this comparison is intended as an indicative benchmark rather than a strictly equivalent performance ranking. Experimental conditions explicitly reported in the cited studies are included in Table 6.

Under the present experimental conditions, H-BC showed a Langmuir-derived apparent retention capacity of 77.44 mg/g at 55 °C. This value was higher than those of the selected benchmark materials listed in Table 6; however, the comparison should be interpreted cautiously as the reported capacities were obtained under different experimental conditions. Therefore, the internal comparison between H-BC and BC under identical conditions remains the primary evidence for the beneficial effect of H_2_O_2_ functionalization.

### 2.4. Apparent Thermodynamic Assessment of Cr(VI)-Derived Retention on Biochars

To provide a comparative energetic interpretation of Cr(VI)-derived retention, apparent thermodynamic indicators, including ΔG, ΔH, and ΔS, were estimated from the fitted isotherm constants. As Cr(VI) uptake involved concurrent adsorption and partial reduction, these parameters are used as apparent indicators of temperature-dependent retention behavior rather than rigorous state functions for a fully reversible equilibrium system. In addition, the Langmuir constant K_L_ retained units of L/mg and was not converted to a dimensionless standard-state equilibrium constant K°. This interpretation follows the caution raised by Lima et al. [31] that thermodynamic parameters derived from non-dimensionless fitted constants should not be treated as rigorous adsorption thermodynamic quantities. Accordingly, the 55 °C condition should be understood as an accelerated upper-temperature point for comparative apparent retention assessment, rather than as a field-representative environmental temperature [35].

The positive apparent ΔH values should be interpreted cautiously. They indicate that the overall Cr(VI)-derived retention–reduction process became more favorable at elevated temperature, but they do not demonstrate that adsorption alone was endothermic. The thermal enhancement may reflect several coupled contributions, including improved mass transfer, partial dehydration of chromate oxyanions, stronger access to OFG-rich binding sites, and activation-energy-demanding interfacial Cr(VI) reduction on selected surface domains [5,17,28]. H_2_O_2_ modification reduced the apparent enthalpy change from 92.21 kJ/mol for BC to 5.58 kJ/mol for H-BC, suggesting that oxidative functionalization lowered the apparent energetic demand of the coupled retention–reduction process. However, the BC-derived apparent ΔH should be viewed with particular caution as it was estimated from only three temperature points and the apparent ΔG variation did not show a robust linear temperature dependence.

Similarly, the apparent ΔS values are not used here to infer a physically rigorous adsorption entropy change, particularly as the previously large BC-associated ΔS may arise from the use of fitted constants that do not satisfy the strict requirements for thermodynamic equilibrium constants [5]. Collectively, the apparent thermodynamic trends are retained only as comparative indicators of temperature-enhanced Cr(VI)-derived retention under the tested conditions and should not be used to separate adsorption entropy, adsorption enthalpy, reduction-related activation effects, or mass-transfer contributions [5,17].

The adsorption, electrokinetic, and spectroscopic results indicate that the enhanced Cr(VI)-derived retention by H-BC arises from reaction-coupled interfacial processes rather than increased electrostatic attraction to chromate oxyanions. Under the near-neutral experimental conditions, the negatively charged H-BC surface makes simple Coulombic attraction an unlikely dominant mechanism. Instead, chemically heterogeneous oxygenated domains may promote short-range, non-electrostatic interactions between Cr(VI) species and residual C–O/O–H moieties or conjugated carbon regions. These interactions may facilitate partial electron transfer to Cr(VI), whereas carbonyl- and carboxylate-containing groups may coordinate and stabilize the resulting surface-associated Cr(III) [3,6,7]. The FTIR changes in the O–H and C–O regions [3,6,7], together with the higher surface Cr(III) fraction observed by XPS, support this reaction-coupled retention pathway [6,7]. XPS characterizes surface-bound chromium on the recovered biochar and does not provide a system-level Cr mass balance. Therefore, the subsequent soil-phase experiments are interpreted as operationally filtered Cr(VI) mobility tests rather than measurements of total chromium release or complete chromium immobilization. A complete chromium balance would require concurrent determination of Cr(VI)aq, dissolved Cr(III), filtered and unfiltered total Cr in leachates/extracts, and solid-phase chromium fractions. This interpretation is further supported by the DFT-based electronic-structure analysis presented in Section 2.7, which indicates enhanced interfacial electronic responsiveness of H-BC after H_2_O_2_ functionalization [36,37].

### 2.5. Dynamic Leaching of Operationally Filtered Cr(VI) in Soil Columns

**Figure 7:**
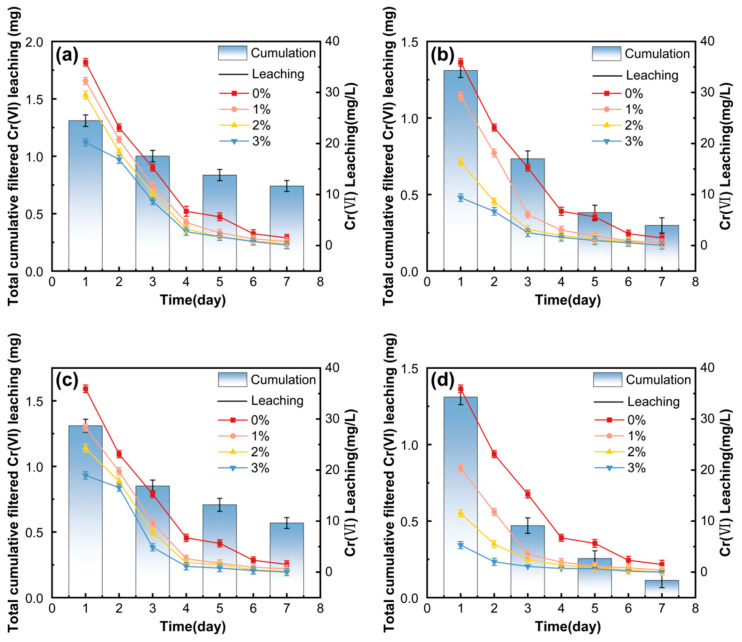
Dynamic column leaching profiles of operationally filtered Cr(VI) under daily intermittent DI-water flushing over a 7-day period. The panels compare the anti-leaching efficacy of pristine biochar (BC) and H_2_O_2_-engineered biochar (H-BC) across different spatial configurations: (**a**) BC with stratified interception; (**b**) BC with homogeneous mixing; (**c**) H-BC with stratified interception; and (**d**) H-BC with homogeneous mixing. Line graphs show daily filtered effluent Cr(VI) concentrations at varying amendment dosages (0–3 wt%), while bar charts show total cumulative filtered Cr(VI) leaching over the 7-day flushing period. The apparent column-retained Cr(VI) inventory was estimated as M_ret,app_ = M_0_ − M_cum_, where M_0_ was 3.046 mg per column. Data are presented as mean ± SD from triplicate measurements, and error bars represent standard deviations.

To simulate hydrodynamic flushing and evaluate the resistance of immobilized Cr(VI) to leaching, dynamic 7-day soil column assays were conducted using previously aged Cr(VI)-spiked sandy loam soil prepared by K_2_Cr_2_O_7_ addition, with an initial Cr(VI) loading of 15.23 mg/kg. As shown in the temporal leaching profiles (Figure 7), all experimental treatments exhibited a pronounced first-flush effect, with the highest Cr(VI) release occurring within the initial 48 h. The unamended control soil showed a pronounced first-flush release, with 0.862 mg of filtered Cr(VI) released on Day 1 and a cumulative filtered Cr(VI) release of 1.310 mg over 7 days (Figure 7b). This trend is consistent with the high mobility of Cr(VI) oxyanions and their limited retention by the native soil under hydraulic flushing [2,38]. Conversely, the incorporation of carbonaceous amendments markedly reduced the release of operationally filtered Cr(VI). The 3% (*w*/*w*) H-BC treatment showed the strongest suppression of filtered Cr(VI) leaching; by Day 5, the filtered effluent Cr(VI) concentration decreased to 0.51 mg/L, corresponding to an 87.5% reduction relative to the unamended control. Over the 7-day column test, cumulative filtered Cr(VI) release from the 3% H-BC treatment was limited to 0.113 mg, equivalent to a 91.4% filtered Cr(VI)-based retention efficiency. Under the same dosage, pristine BC allowed a higher cumulative filtered Cr(VI) release of 0.298 mg, indicating that oxidative functionalization improved resistance to filtered Cr(VI) transport under hydraulic flushing [2].

To clarify the cumulative leaching parameter, the daily filtered Cr(VI) loads were further expressed as total cumulative filtered Cr(VI) leaching over the 7-day flushing period and compared with the apparent column-retained Cr(VI) inventory [39,40]. Based on the initial Cr(VI) inventory of 3.046 mg per column, M_ret,app_ was estimated as M_0_ − M_cum_. At the 3% amendment level, total cumulative filtered Cr(VI) leaching decreased from 1.310 mg in the unamended control to 0.741 mg for stratified BC, 0.298 mg for homogeneous BC, 0.569 mg for stratified H-BC, and 0.240 mg for homogeneous H-BC. Correspondingly, M_ret,app_ increased from 1.736 mg in the control to 2.305, 2.748, 2.477, and 2.806 mg, respectively. This comparison indicates that homogeneous H-BC most effectively reduced cumulative filtered Cr(VI) leaching while maintaining the largest apparent Cr(VI) inventory within the soil column [38,39].

These leaching and apparent-retention results should be interpreted within the operational definition of the analytical protocol. As effluents were passed through 0.45-μm membranes before DPC determination, the measured endpoint represents filtered Cr(VI), rather than total chromium release. Therefore, a decrease in DPC-detectable Cr(VI) may reflect adsorption onto soil/biochar surfaces, partial reduction to Cr(III), retention of chromium-bearing colloids or particles during filtration, or a combination of these processes. A complete leaching-scale chromium balance would require parallel analysis of Cr(VI), dissolved Cr(III), and filtered/unfiltered acid-digested total Cr in the effluents, together with solid-phase Cr quantification after column operation [38,41,42].

For field-oriented assessment, the present DI-water column leaching test should therefore be considered a mobility-screening experiment rather than a regulatory leachability test. As BC/H-BC is intended as an in situ soil amendment and is unlikely to be recovered after application, regeneration is less relevant for soil remediation than post-stabilization leaching resistance. Future validation should include USEPA TCLP-type acetic acid leaching, together with Cr(VI), dissolved Cr(III), filtered/unfiltered total Cr, and solid-phase Cr fractionation.

Within this operationally defined framework, the stronger suppression of filtered Cr(VI) release by H-BC is consistent with enrichment of oxygen-containing functional groups (OFGs) [5,12]. These OFGs may contribute to reaction-coupled interfacial retention through non-electrostatic association, partial reduction, and stabilization of surface-associated Cr(III) [17]. Crucially, the architectural configuration of the amendment profoundly influenced the remediation outcomes. The homogeneous incorporation of H-BC substantially outperformed the stratified (layered) arrangement. This disparity indicates that uniform spatial dispersion maximizes the interfacial contact probability along the entire percolation pathway. Conversely, a localized reactive interlayer (stratification) inevitably suffers from premature site saturation and preferential flow bypass, thereby compromising overall efficacy [2,39]. The column results further indicate that homogeneous amendment distribution improves contact between migrating Cr(VI) species and H-BC reactive sites along the percolation pathway under the tested laboratory conditions. Field-transferable evaluation of this placement strategy will require future column studies with packed bulk density, true pore volume, pore-volume-normalized flow, saturation/residence-time characterization, leachate pH/Eh monitoring, and paired Cr(VI), Cr(III), filtered/unfiltered total-Cr mass recovery [2,11].

### 2.6. Immobilization of Cr(VI) in Contaminated Soil Under Wet-Dry Cycles

**Figure 8:**
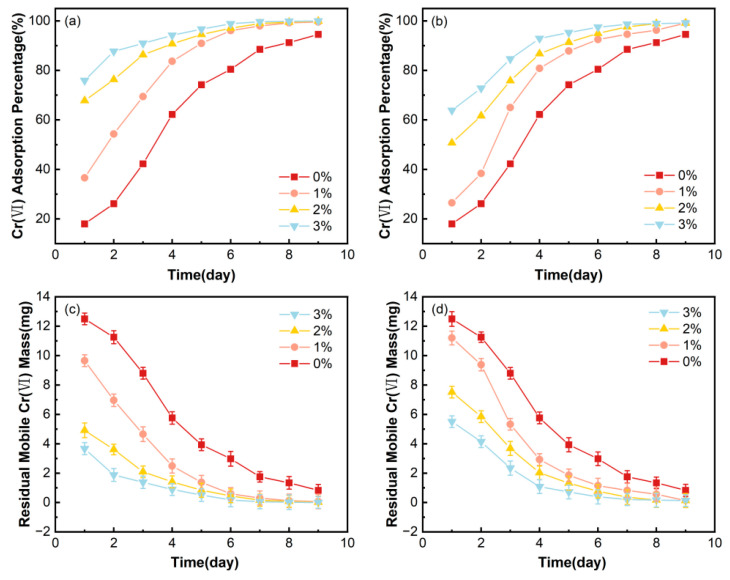
Temporal attenuation dynamics of filtered water-extractable Cr(VI) in contaminated soil under accelerated wet–dry (W–D) cycling. The calculated percentages represent filtered-Cr(VI)-based attenuation of the water-extractable fraction and should not be interpreted as complete total-Cr immobilization. The top panels display the time-dependent attenuation percentages for soil matrices amended with varying dosages (0–3 wt%) of (**a**) H_2_O_2_-engineered biochar (H-BC) and (**b**) pristine biochar (BC). The bottom panels show the corresponding temporal profiles of residual filtered water-extractable Cr(VI) for the (**c**) H-BC and (**d**) BC treatments. Data are presented as mean ± SD from triplicate measurements, and error bars represent standard deviations.

To simulate climatic fluctuations and evaluate the stability of the mobile Cr(VI) pool, 9-day soil incubation assays were subjected to accelerated wet–dry (W–D) cycling. As shown in Figure 8c,d, the unamended control soil also exhibited a time-dependent decrease in filtered water-extractable Cr(VI), reaching 0.829 mg/L by day 9. This response should be interpreted as baseline attenuation of the operationally filtered Cr(VI) fraction rather than intrinsic immobilization of total chromium. Although the low-SOM soil has limited natural reductive buffering capacity, W–D cycling may still decrease Cr(VI) extractability through mineral sorption, limited reduction by residual organic matter or redox-sensitive mineral phases, pore-scale aging, and changes in water-extractable fractions [2,12]. The addition of carbonaceous amendments accelerated Cr(VI) attenuation in a dose-responsive manner [2,43]. By day 5, filtered water-extractable Cr(VI) concentrations in the 1%, 2%, and 3% H-BC treatments decreased to 1.38, 0.83, and 0.51 mg/L, respectively, compared with 3.93 mg/L in the control. The 2% H-BC treatment achieved 86.29% immobilization within 3 days, whereas the same level of attenuation required 5 days in the BC treatment. By day 9, the 3% H-BC treatment reduced filtered water-extractable Cr(VI) to 0.0025 mg/L, corresponding to a filtered-Cr(VI)-based immobilization efficiency of 99.98% [28].

As in the column-leaching experiment, the W–D aging endpoint is operationally defined by 0.45-μm filtration followed by DPC-based Cr(VI) determination. The results therefore demonstrate suppression of the filtered, water-extractable Cr(VI) fraction rather than total chromium immobilization [44,45]. This distinction is important because reduced Cr(III), colloidal Cr, and chromium-bearing fine particles may still contribute to chromium redistribution under changing redox, hydrodynamic, or acidic leaching conditions. Accordingly, future W–D aging studies should combine Cr(VI)-specific analysis with non-cycled controls, pH/Eh monitoring, dissolved Cr(III), filtered/unfiltered total Cr, sequential solid-phase extraction, and TCLP-type acetic acid leaching to determine whether the attenuated Cr fraction remains stable under field-relevant leaching stress [45,46,47,48,49,50].

Mechanistically, the faster attenuation of filtered water-extractable Cr(VI) in the H-BC-amended soil may arise from the coupling between an OFG-enriched surface and alternating hydration states. During the humidification phase, continuous pore-water films can facilitate advective–diffusive transport of Cr(VI) oxyanions toward reactive oxygenated groups on the H-BC interface, increasing opportunities for short-range interfacial contact [1,5,12]. During drying, capillary concentration and shortened water films may enhance contact between Cr(VI) species and biochar surfaces, thereby favoring closer interfacial contact and reaction-coupled retention [15]. Therefore, the W–D results suggest that H-BC can suppress the filtered mobile Cr(VI) fraction under cyclic hygrothermal stress, although total-Cr redistribution should be further assessed using paired filtered and unfiltered analyses [2,28].

To further support practical translation, future studies should benchmark H-BC against commercial activated carbon and other widely used engineered remediation media under standardized conditions, including identical pH, adsorbent dosage, initial Cr(VI) concentration, contact time, temperature, competing-ion composition, and real-water or soil-matrix backgrounds [24,39,40]. These standardized comparisons would further support application-specific evaluation of H-BC. Reuse stability and cost-effectiveness are more relevant to recoverable water-treatment configurations [28,39,40], whereas in situ soil application should be assessed mainly through post-stabilization leaching resistance and chromium-speciation stability, as discussed above [39,40].

### 2.7. DFT-Based Electronic-Structure Evidence for the Enhanced Reactivity of H-BC

**Figure 9:**
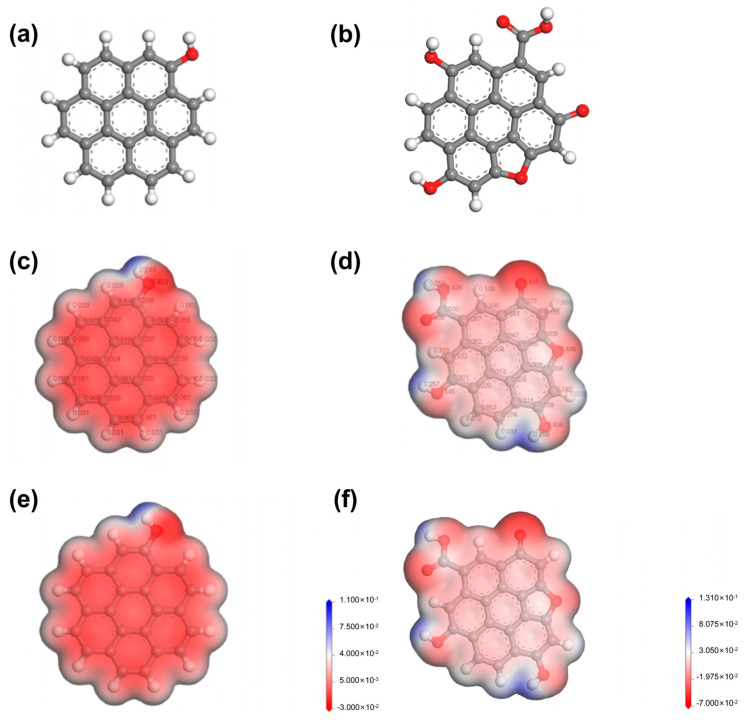
DFT-optimized molecular structures, electrostatic potential surfaces, and electron-density distributions of pristine biochar (BC) and H_2_O_2_-functionalized biochar (H-BC). Panels (**a**,**c**,**e**) correspond to BC, showing the optimized molecular structure, electrostatic potential surface, and electron-density distribution, respectively; panels (**b**,**d**,**f**) correspond to H-BC, showing the corresponding electronic-structure features after oxidative functionalization. The results indicate increased surface polarity and altered electronic distribution in H-BC, consistent with enhanced electronic responsiveness of the functionalized fragment model.

To further support the experimentally inferred adsorption-coupled reduction mechanism, DFT calculations were performed using representative BC and H-BC molecular fragments. As shown in Figure 9, the pristine BC model was represented by an aromatic carbon cluster with limited oxygen-containing edge sites, whereas the H-BC model contained additional hydroxyl, carbonyl, and carboxyl-type groups to reflect the oxidative functionalization induced by H_2_O_2_ treatment. This simplified cluster-model strategy was used to compare the electronic-structure response of BC and H-BC rather than to fully reproduce the structural heterogeneity of the bulk biochar matrix, following recent molecular-simulation approaches used for biochar systems [36,37].

The molecular electrostatic potential and electron-density distributions showed that H_2_O_2_ functionalization generated a more polar and spatially heterogeneous surface on H-BC. These localized polar domains are consistent with the experimentally observed enrichment of oxygen-containing functional groups by SEM–EDS, FTIR, and XPS. More importantly, the frontier molecular orbital analysis revealed a clear decrease in the HOMO–LUMO energy gap from 2.6615 eV for BC to 1.7482 eV for H-BC (Table 7). As a smaller frontier orbital gap generally indicates higher electronic responsiveness, this result suggests that the oxidatively functionalized H-BC fragment may have a stronger tendency for interfacial electronic polarization [36,37].

These DFT results are therefore interpreted as qualitative electronic-structure support rather than direct evidence for chromate adsorption, charge transfer, or the Cr(VI)-to-Cr(III) reaction pathway. The increased polarity and reduced HOMO–LUMO gap are consistent with a more electronically responsive H-BC interface, whereas the adsorption-coupled reduction mechanism is evaluated mainly from the combined experimental evidence, including FTIR-indicated functional group participation and XPS Cr 2p surface speciation. Future DFT studies should construct explicit HCrO_4_^−^/CrO_4_^2−^ adsorption complexes on BC and H-BC models and evaluate adsorption energies, charge redistribution, spin/electronic states, and possible reaction pathways [3,6,7].

## 3. Materials and Methods

### 3.1. Soil Sampling and Preparation of Cr(VI)-Contaminated Soil

Potassium dichromate (K_2_Cr_2_O_7_) and hydrogen peroxide (H_2_O_2_, 30% *w*/*w*), both of analytical purity, were procured from Shengshi Standard Material Co., Ltd. (Cangzhou, China) and Henan Huize Bioengineering Co., Ltd. (Xinxiang, China), respectively. Supplementary chemicals utilized throughout the experimental phases were exclusively of analytical grade and were applied as received, negating the need for secondary purification, a standard stringent protocol matching recent dual-modification biochar and polymer composite studies [4]. To establish a representative pedological matrix, superficial soil specimens (0–20 cm depth profile) were excavated from a characteristic semi-arid ecosystem located in Songpan County, Sichuan Province, China. The bulk samples were naturally desiccated at ambient temperature, mechanically pulverized, and subsequently passed through a 2 mm stainless-steel sieve to meticulously eliminate coarse lithic fragments, macroscopic root detritus, and extraneous debris [2]. Physicochemical profiling categorized the substrate as a typical sandy loam. The soil reaction pH was determined potentiometrically in a 1:2.5 (*w*/*v*) air-dried soil/deionized-water suspension after equilibration using a calibrated glass-electrode pH meter. Soil organic matter (SOM) was determined from soil organic carbon measured by K_2_Cr_2_O_7_–H_2_SO_4_ wet oxidation with external heating, followed by back-titration of the residual oxidant with ferrous ammonium sulfate; SOC was converted to SOM using the van Bemmelen factor of 1.724. The soil exhibited a mildly acidic pH of 6.5 and a severely depleted SOM content of approximately 0.5%, consistent with the oligotrophic characteristics of the selected Songpan sandy loam. To construct a reproducible Cr(VI)-spiked soil model while distinguishing the experimentally introduced Cr(VI) from the native chromium background, the K_2_Cr_2_O_7_ stock solution was prepared at 43.08 g/L K_2_Cr_2_O_7_, equivalent to 15.23 g/L as Cr(VI). A 10 mL aliquot of this stock solution was transferred into a 1000 mL volumetric flask and diluted to volume with deionized water. The diluted K_2_Cr_2_O_7_ solution was gradually added to 10 kg of screened Songpan sandy loam, corresponding to a soil/solution ratio of 10 kg/L, equivalent to 10 g/mL (*w*/*v*). The mixture was mechanically homogenized to improve the spatial uniformity of Cr(VI) distribution, resulting in a target initial Cr(VI) loading of 15.23 mg/kg. This loading was selected to establish a controlled Cr(VI)-impaired sandy-loam matrix for subsequent leaching and aging experiments while preserving the low-SOM characteristics of the native soil [51,52]. The native mildly acidic soil pH was not artificially adjusted, because the study aimed to evaluate Cr(VI) mobility and biochar-assisted immobilization under the inherent buffering conditions of the selected Songpan soil. The soil/solution ratio was chosen to provide sufficient wetting for homogeneous K_2_Cr_2_O_7_ distribution without producing an excessively waterlogged slurry. The spiked soil was aged for 72 h at 25 °C under natural-moisture conditions to allow short-term redistribution of soluble chromate species within the soil matrix [53]. The aged soil was then dried at 60 °C, gently disaggregated, and stored for subsequent column leaching, wet–dry aging, and amendment-placement experiments [1,54]. The drying temperature was selected to remove moisture and improve handling and column-packing reproducibility while avoiding stronger thermal treatment that could substantially alter soil organic matter or chromium speciation.

### 3.2. Preparation and H_2_O_2_ Modification of Biochar

Concurrently with the formulation of the heavy-metal-impaired soil matrix, the carbonaceous remediation platforms were prepared from industrial wood-processing residues, mainly waste wood chips and sawdust generated during wood processing [55,56]. These lignocellulosic residues were repeatedly rinsed to remove superficial contaminants, air-dried at ambient temperature, mechanically comminuted, and fractionated through a standard test sieve to obtain a homogeneous precursor fraction [10].Subsequent carbonization of the fractionated biomass was executed within a quartz tube furnace. To establish a strictly anoxic environment, a continuous nitrogen (N_2_) purge was applied while the system was heated at a linear ramp rate of 5 °C/min. The peak pyrolytic temperature was set at 700 °C and held isothermally for a duration of 4 h. Upon completion of the thermal treatment, the pristine biochar (BC) was allowed to cool passively to ambient temperature (~25 °C) under an uninterrupted N_2_ flow to prevent premature auto-oxidation, aligning with optimal pyrolytic protocols for biomass-derived polymers [9]. To achieve targeted surface functionalization, a wet-chemical oxidation protocol was employed. Specifically, 150 g of the synthesized BC was dispersed into 1000 mL of aqueous H_2_O_2_ solution (7.5% *w*/*w*; initial pH 4.37), thereby establishing a solid-to-liquid ratio of 150 g/L. The suspension was magnetically stirred at 25 °C for 3 h to promote oxidative etching and oxygen-containing functional group enrichment on the carbon framework [3,4]. After oxidation, the functionalized solid phase was collected by vacuum filtration and repeatedly rinsed with ultrapure water until the eluate reached neutral pH, which served as the operational final washing endpoint. The purified material was subsequently dried in a forced-air convection oven at 80 °C, yielding the final H_2_O_2_-engineered woody biochar (H-BC) [8].

The key preparation parameters for H-BC were N_2_-assisted pyrolysis at 700 °C for 4 h with a heating rate of 5 °C/min, followed by oxidative functionalization of 150 g BC in 1000 mL of 7.5% H_2_O_2_ solution with an initial pH of 4.37 at 25 °C for 3 h, repeated washing to neutral pH, and drying at 80 °C [8,10].

These preparation conditions were selected to balance carbon-framework stability, surface oxidation, and post-treatment material stability. N_2_-assisted pyrolysis at 700 °C for 4 h was used to obtain a stable woody biochar matrix with a carbonized framework suitable for subsequent wet oxidation [57]. The H_2_O_2_ concentration, solid-to-liquid ratio, reaction temperature, and oxidation duration were selected to promote oxygen-containing functional group enrichment and local oxidative etching under mild aqueous conditions, while limiting excessive carbon loss or uncontrolled oxidation [58]. Repeated washing to neutral pH was performed to remove residual soluble oxidants and acidity, and drying at 80 °C was used to obtain a stable solid H-BC product without applying any additional high-temperature treatment after functionalization.

### 3.3. Analytical Methods

The surface morphology and elemental composition of pristine biochar (BC), H_2_O_2_-modified biochar (H-BC), and Cr-loaded biochar samples were characterized by field-emission scanning electron microscopy coupled with energy-dispersive X-ray spectroscopy (FE-SEM–EDS; Verios XHR SEM, Thermo Fisher Scientific, Brno, Czech Republic). SEM imaging was used to observe surface morphology and pore features, while EDS analysis was used to compare relative elemental compositions, particularly changes in C, O, and Cr contents after H_2_O_2_ modification and Cr(VI) adsorption.

The specific surface area and pore-structure characteristics of BC and H-BC were determined by N_2_ adsorption–desorption analysis using an ASAP 2460 surface area and porosity analyzer (Micromeritics, Norcross, GA, USA). Before measurement, the samples were degassed at 100 °C for 12 h to remove physically adsorbed water and volatile impurities. The N_2_ adsorption–desorption isotherms were then collected at 77 K. The specific surface area was calculated using the Brunauer–Emmett–Teller (BET) method, while the total pore volume and pore-size distribution were derived from the N_2_ adsorption data. These measurements were used to quantitatively evaluate the textural changes induced by H_2_O_2_ oxidative functionalization.

Fourier transform infrared spectroscopy (FTIR; Nicolet iS50, Thermo Fisher Scientific, Waltham, MA, USA) was performed to identify the surface functional groups of the biochar samples and to monitor their changes after Cr(VI) adsorption. The spectra were recorded over the wavenumber range of 4000–400 cm^−1^. Particular attention was paid to the absorption regions associated with surface hydroxyl groups, carbonyl/aromatic skeletal vibrations, and C–O/C–O–C moieties. These spectral features were used to evaluate the evolution of oxygen-containing functional groups and their possible involvement in Cr(VI) binding.

X-ray photoelectron spectroscopy (XPS; ESCALAB, Thermo Fisher Scientific, Waltham, MA, USA) was used to determine the surface elemental composition, chemical bonding states, and chromium speciation of the biochar samples after Cr(VI) adsorption. High-resolution C 1s, O 1s, and Cr 2p spectra were collected to analyze oxygenated carbon species, surface oxygen functionalities, and the relative proportions of Cr(VI) and Cr(III). Binding energies were calibrated against the carbon-framework C–C peak at 284.8 eV.

The electrokinetic properties of BC and H-BC were evaluated by zeta-potential measurements using a Nano-ZS analyzer (Malvern Zetasizer Nano ZS, Malvern Panalytical Ltd., Malvern, UK). Biochar suspensions were prepared by dispersing the samples in deionized water and thoroughly homogenizing them before measurement. The suspension pH was adjusted to 6.5, and the measurements were conducted at 25 °C. The recorded conductivities were 0.024 ± 0.002 mS/cm for BC and 0.039 ± 0.003 mS/cm for H-BC. As no background electrolyte was intentionally added, the reported zeta potentials are interpreted as apparent electrokinetic potentials under these defined aqueous suspension conditions [21,22,23]. The point of zero charge (pH_PZC_) was determined using an acid–base titration method [21,23]. Briefly, biochar suspensions were adjusted to a series of initial pH values using dilute HCl or NaOH solutions and then equilibrated under constant agitation. After stabilization, the final pH values were recorded. The pH_PZC_ was identified as the point at which the difference between the final and initial pH values approached zero. The combined zeta-potential and pH_pzc_ results were used to evaluate surface electronegativity, acidic surface character, and the ionization behavior of oxygen-containing functional groups.

### 3.4. Batch Adsorption Experiments

To systematically evaluate the Cr(VI) sequestration capacity, batch adsorption assays were executed by dispersing 0.5 g of the respective biochar into 100 mL of Cr(VI) solutions within 250 mL Erlenmeyer flasks. The mixtures were subjected to continuous agitation at 150 rpm within a thermostatic orbital shaker to maintain homogeneous solid–liquid contact. All trials were performed in independent triplicates to ensure statistical reproducibility, reporting the arithmetic means [4,28,59]. For the acquisition of isothermal trajectories, the initial Cr(VI) concentrations (C_0_) were systematically varied from 17.67 to 106.06 mg/L. The suspensions were agitated for 6 h at 25, 40, and 55 °C to obtain temperature-dependent isotherm datasets. Unless otherwise specified for pH-dependency trials, the initial solution pH was adjusted to 6.0 ± 0.1 using 0.1 mol/L HCl or 0.1 mol/L NaOH (analytical grade; SENHOPE, Xiamen, China) [1,26].

The concentration range of 17.67–106.06 mg/L was selected to cover low-to-high Cr(VI) loading conditions suitable for kinetic evaluation and apparent isotherm fitting at a fixed adsorbent concentration of 5.0 g/L. The adsorbent dosage of 0.5 g in 100 mL solution was used to ensure sufficient solid–liquid contact while maintaining measurable residual Cr(VI) concentrations across the tested range. The initial solution pH of 6.0 ± 0.1 was selected to represent a mildly acidic to near-neutral aqueous condition related to the soil matrix and to avoid strongly acidic conditions under which chromate retention by biochar may be dominated by protonation-enhanced electrostatic attraction. The 6 h contact time exceeded the dynamic equilibrium period observed in the kinetic experiments, allowing the isotherm samples to approach equilibrium under the tested conditions. The temperature series of 25, 40, and 55 °C was used to evaluate apparent temperature-dependent retention behavior. In this design, 55 °C was included as an accelerated upper-temperature point to amplify temperature-related changes in the coupled retention–reduction process, rather than as a field-representative soil temperature.

To elucidate the time-dependent mass transfer behavior, kinetic assays were conducted at 25 °C utilizing a fixed *C*_0_ of 106.06 mg/L. Aliquots (10 mL) were extracted at predefined temporal intervals spanning from 0 to 360 min. To halt the reaction and separate the solid phase, the aliquots were immediately passed through 0.45 μm syringe filters. The residual aqueous Cr(VI) concentrations were then quantified spectrophotometrically at λ = 540 nm following complexation with 1,5-diphenylcarbazide (DPC; analytical grade; UNICO (Shanghai) Instruments Co., Ltd., Shanghai, China) [27,59,60]. For reproducibility, the adsorption system consisted of 0.5 g biochar and 100 mL Cr(VI) solution in 250 mL Erlenmeyer flasks under 150 rpm agitation; the initial solution pH was adjusted to 6.0 ± 0.1 using 0.1 mol/L HCl or 0.1 mol/L NaOH, equilibrium tests were conducted for 6 h at 25, 40, or 55 °C, and aliquots were filtered through 0.45-μm membranes before DPC analysis at 540 nm [28,61]. This Cr(VI)-specific analytical protocol did not include dissolved TOC measurement or aqueous Cr(III)/total-Cr speciation of the filtrates.

The transient (q_t_) and equilibrium (q_e_) solid-phase concentrations (mg/g) were determined via mass balance principles, expressed as Equations (1) and (2) [28,61].

(1) $$q_{e}=\frac{\left(C_{0}-C_{e}\right)\times V}{m}$$

(2) $$q_{t}=\frac{\left(C_{0}-C_{t}\right)\times V}{m}$$

where *C*_e_ and *C*_t_ (mg/L) denote the residual Cr(VI) concentrations at equilibrium and at any given time t, respectively; *V* designates the solution volume (0.1 L), and m represents the applied biochar mass (0.5 g).

To describe the post-contact relationship between residual Cr(VI) concentration and Cr(VI)-derived retention on the solid, the experimental datasets were fitted using the Langmuir (Equation (3)) and Freundlich (Equation (4)) equations as apparent isotherm models [15,30,61,62,63]:

(3) $$q_{e}=\frac{q_{m}K_{L}C_{e}}{1+K_{L}C_{e}}$$

(4) $$q_{e}=K_{F}C_{e}^{1/n}$$

Here, q_m_ denotes the Langmuir-fitted/extrapolated apparent retention capacity, K_L_ (L/mg) is the Langmuir affinity constant, K_F_ represents the Freundlich capacity indicator, and n describes the fitting intensity and apparent surface heterogeneity. Under the tested V/m ratio and C_0_ range, q_m_ is a model-derived parameter for comparative interpretation and should not be interpreted as the maximum directly measured q_e_ or as an experimentally observed saturation capacity [29,30,62].

For the apparent thermodynamic assessment, K_L_ was retained in its fitted unit of L/mg and was not converted to a dimensionless standard-state equilibrium constant K°. Accordingly, the derived ΔG, ΔH, and ΔS values are reported as apparent comparative indicators of temperature-dependent Cr(VI)-derived retention. These values are not used as rigorous standard-state thermodynamic quantities or as direct evidence for adsorption enthalpy, adsorption entropy, or a fully reversible equilibrium process.

Concurrently, the temporal kinetic trajectories were interpreted by applying the pseudo-first-order (PFO, Equation (5)) and pseudo-second-order (PSO, Equation (6)) rate models to compare empirical kinetic patterns and apparent rate parameters [15,26]:

(5) $$q_{t}=q_{e}\left(1-e^{-k_{1}t}\right)$$

(6) $$q_{t}=\frac{k_{2}q_{e}^{2}t}{1+k_{2}q_{e}t}$$

Herein, k_1_ (min^−1^) and k_2_ [g/(mg·min)] correspond to the specific rate constants for the PFO and PSO models, respectively.

To compare empirical fitting performance beyond *R*^2^, the root mean square error (RMSE), Akaike information criterion (AIC), and Bayesian information criterion (BIC) were calculated from the residuals between the experimental and model-fitted q_t_ values. The residual sum of squares (RSS) was first calculated according to Equation (7):

(7) $$RSS=\sum_{i=1}^{n}{\left(q_{t,obs,i}-q_{t,fit,i}\right)}^{2}$$

The RMSE, AIC, and BIC were then calculated according to Equations (8)–(10), respectively:

(8) $$RMSE={\left(\frac{RSS}{n}\right)}^{1/2}$$

(9) $$AIC=n\ln(\frac{RSS}{n})+2p$$

(10) $$BIC=n\ln(\frac{RSS}{n})+p\ln(n)$$

where q_t,obs,i_ and q_t,fit,i_ are the experimental and model-fitted adsorption capacities at the i-th sampling time, respectively; *n* is the number of observations; and *p* is the number of fitted model parameters. For both the PFO and PSO models, *p* = 2, corresponding to the fitted equilibrium capacity and the apparent rate constant.

To further delineate the sequential mass-transfer pathways and evaluate diffusion-related contributions, the Weber–Morris intra-particle diffusion (IPD) model (Equation (11)) was utilized via piecewise linear regression [26,27,40]:

(11) $$q_{t}=k_{pi}t^{1/2}+C_{i}$$

Within this framework, the multilinear adsorption trajectory is characteristically partitioned into three sequential stages: rapid external boundary layer transport (film diffusion), progressive intra-particle diffusion within the porous architecture, and the ultimate dynamic equilibrium. The parameter k_pi_ denotes the stage-specific diffusion rate constant, while the intercept *C*_i_ correlates directly with the thickness of the hydrodynamic boundary layer. Notably, a non-zero *C*_i_ signifies that the internal pore diffusion is not the exclusive kinetic constraint; rather, the macroscopic adsorption rate is governed synergistically by both boundary layer resistance and intra-particle mass transfer hindrances [27,40].

The Boyd model was further used to distinguish whether intra-particle diffusion was the sole rate-controlling step or whether external film diffusion contributed to the adsorption process [64]. The fractional adsorption at time t was calculated according to Equation (12):

(12) $$F=\frac{q_{t}}{q_{e}}$$

where F_t_ is the fractional adsorption at time t, and q_t_ and q_e_ are the adsorption capacities at time t and equilibrium, respectively. The Boyd parameter *B*_t_ was then estimated using Equation (13):

(13) $$B_{t}=-0.4977-\ln(1-F)$$

The plots of *B*_t_ versus t were fitted by linear regression. A Boyd plot passing through the origin indicates intra-particle-diffusion control, whereas deviation from the origin suggests the involvement of external film diffusion.

### 3.5. Soil Column Leaching Experiments

To dynamically assess the vertical migration and retention profiles of Cr(VI) under controlled hydrodynamic flushing, laboratory-scale soil column assays were systematically conducted [2,38,39]. The cylindrical columns had dimensions of 7 cm × 14 cm, and 1 mm-diameter drainage holes were present at the bottom of each column to allow effluent discharge. Each experimental column was packed with 200 g of the previously aged Cr(VI)-spiked soil. The matrices were amended with either pristine biochar (BC) or H_2_O_2_-modified biochar (H-BC) at 0%, 1%, 2%, or 3% (*w*/*w*). To evaluate the influence of spatial distribution, the amendments were applied as either homogeneous incorporation throughout the 200 g soil matrix or stratified interception as a localized reactive interlayer between two 100 g contaminated-soil layers [9,39]. Each packed column received 24 mL of deionized (DI) water with an initial pH of approximately 7.23 every 24 h for 7 consecutive days, corresponding to a nominal 24 h flushing interval. DI water was selected to isolate the immobilization effect of BC/H-BC by minimizing carbonate-driven pH shifts, ionic-strength variation, and additional dissolved inorganic carbon inputs that could independently alter Cr(VI)/Cr(III) speciation and soil–biochar surface charge. The resulting effluents were quantitatively recovered on a daily basis. For the Cr(VI)-specific endpoint, a 10 mL aliquot of each leachate was passed through a 0.45-μm membrane by vacuum filtration before analysis. The Cr(VI) concentration in the filtrate was quantified spectrophotometrically using the standard 1,5-diphenylcarbazide (DPC) colorimetric assay. Therefore, the column results represent operationally filtered Cr(VI) leaching; the results do not represent total Cr leaching or a closed chromium mass balance. Total Cr, dissolved Cr(III), and chromium associated with suspended particles retained by the membrane were not determined in this experimental design. Accordingly, the column dataset was used for comparative evaluation of filtered-Cr(VI) transport suppression under fixed laboratory conditions and was not intended for pore-volume-normalized breakthrough analysis, complete total-Cr mass recovery, or field-scale hydraulic transport modeling. The cumulative mass of leached filtered Cr(VI) (M_cum_, mg) throughout the 7-day flushing duration was calculated by summing the daily filtered-Cr(VI) loads according to Equation (14):

(14) $$M_{cum}=\sum_{i=1}^{n}\left(C_{i}\times V_{i}\right)$$

where *C*_i_ (mg/L) denotes the filtered Cr(VI) concentration detected in the effluent on day i, *V* (L) represents the constant daily leachate volume (0.024 L), and *n* corresponds to the total number of elution cycles (*n* = 7). For comparison with cumulative leaching, the apparent column-retained Cr(VI) inventory was estimated from the difference between the initial Cr(VI) inventory and the total cumulative filtered Cr(VI) leaching:

(15) $$M_{ret,app}=M_{0}-M_{cum}$$

where *M*_ret,app_ is the apparent column-retained Cr(VI) inventory, *M*_0_ is the initial Cr(VI) inventory in each soil column, and *M*_cum_ is the total cumulative filtered Cr(VI) leaching calculated using Equation (14). In this study, *M*_0_ was 3.046 mg, calculated from the initial soil Cr(VI) loading of 15.23 mg/kg and the dry soil mass of 0.200 kg.

Furthermore, the filtered-Cr(VI)-based retention efficiency (η, %) of the biochar interventions against filtered Cr(VI) mobility was evaluated based on Equation (16).

(16) $$\eta =\frac{M_{CK}-M_{Treatment}}{M_{CK}}\times 100\%$$

Here, *M*_CK_ and *M*_Treatment_ signify the cumulative filtered Cr(VI) mass (mg) leached from the unamended control and the biochar-amended soil columns, respectively [1,2,38].

Thus, the column-leaching tests were defined by a fixed soil mass of 200 g, BC/H-BC dosages of 0–3 wt%, homogeneous or stratified amendment placement, 24 mL DI water applied every 24 h for 7 days, daily effluent collection, 0.45-μm filtration, and DPC-based quantification of filtered Cr(VI), rather than total Cr [38,39].

Accordingly, the term “filtered-Cr(VI)-based retention efficiency” is used to describe the DPC-derived endpoint throughout the soil-leaching and W–D aging analyses.

### 3.6. Wet-Dry Aging and Stability Assessment

To evaluate the long-term stabilization efficacy and environmental immobilization stability of the biochar amendments under accelerated weathering, dynamic wet–dry (W–D) cycling assays were implemented [2,11]. Aliquots of the Cr(VI)-spiked soil matrix (200 g each) were homogeneously blended with either the pristine biochar (BC) or the engineered H_2_O_2_-modified variant (H-BC) across a dosage gradient of 0%, 1%, 2%, and 3% (*w*/*w*). To emulate severe climatic fluctuations and hygrothermal stress, the amended matrices were exposed to controlled alternating moisture and thermal regimes, an accelerated aging protocol widely validated in recent environmental and polymeric composite stability evaluations [15,65]. A single 24 h W–D iteration comprise12 h12 h thermal desiccation phase within a forced-air convection oven set at 60 °C, immediately succeeded by a 12 h humidification phase in an environmental chamber maintained at 25 °C with 95% relative humidity. This cyclic weathering protocol was sustained for 9 consecutive iterations, spanning a total of 9 days. To continuously track the immobilization kinetics, representative soil fractions were destructively sampled at 24 h intervals. The highly bioavailable, water-extractable Cr(VI) pool was subsequently mobilized by agitating the sampled soil with deionized (DI) water under constant mechanical shaking. Following extraction, the suspensions were clarified through 0.45-μm membrane filters, and the Cr(VI) concentration in the filtrate was quantified spectrophotometrically at λ = 540 nm using the standard 1,5-diphenylcarbazide (DPC) assay [60]. Accordingly, the W–D aging test quantified filtered, water-extractable Cr(VI), rather than total Cr or chromium carried by non-filtered suspended particles. Therefore, the calculated R_e_ value represents filtered-Cr(VI)-based attenuation during W–D cycling and should not be interpreted as a closed chromium mass balance or complete total-Cr immobilization. The time-dependent immobilization efficiency (*R*_e_, %) was mathematically derived using Equation (17):

(17) $$R_{e}\left(\%\right)=\frac{C_{0}-C_{e}}{C_{0}}\times 100\%$$

where *C*_0_ and *C*_e_ (mg/L) denote the initial and transient filtered water-extractable Cr(VI) concentrations detected in the soil extracts, respectively. To guarantee data robustness and statistical validity, all aging and extraction protocols were executed in independent triplicates, with the empirical results articulated as arithmetic means ± standard deviations (SD).

The W–D aging protocol was defined by 200 g soil per treatment, 0–3 wt% BC/H-BC amendment, nine 24 h cycles consisting of 12 h drying at 60 °C followed by 12 h humidification at 25 °C and 95% relative humidity, 24 h destructive sampling, 0.45-μm filtration, and triplicate DPC-based measurements of filtered Cr(VI) [2,38].

### 3.7. Density Functional Theory Calculations

Density functional theory (DFT) calculations were conducted to provide supplementary molecular-level insight into the effect of H_2_O_2_ functionalization on the electronic structure of biochar. Representative molecular fragments of pristine biochar (BC) and H_2_O_2_-functionalized biochar (H-BC) were constructed using aromatic carbon clusters with hydrogen-saturated edges, following the common practice of using simplified molecular fragments to represent biochar-like carbon domains in theoretical calculations [36,37]. For the H-BC model, hydroxyl, carbonyl, and carboxyl-type oxygen-containing functional groups were introduced at edge or defect sites to represent the surface oxidation induced by H_2_O_2_ treatment. The purpose of these models was to compare the relative electronic reactivity of BC and H-BC rather than to reproduce the full structural complexity of biochar. No explicit HCrO_4_^−^/CrO_4_^2−^ adsorption complex, charge-transfer pathway, spin-state analysis, or reduction transition state was constructed in the present fragment-level calculation.

Geometry optimization was performed at the B3LYP-D3(BJ)/def2-SVP level, followed by single-point energy refinement at the B3LYP-D3(BJ)/def2-TZVP level. The SMD water model was used to approximate the aqueous interfacial environment. Molecular electrostatic potential, electron-density distribution, and frontier molecular orbitals were analyzed to compare the electronic characteristics of BC and H-BC. The HOMO–LUMO energy gap was calculated as Equation (18):

(18) $$\Delta E=E_{LUMO}-E_{HOMO}$$

where E_LUMO_ and E_HOMO_ represent the energies of the lowest unoccupied molecular orbital and the highest occupied molecular orbital, respectively. The calculated energy gap was converted from Hartree to electronvolt using 1 Hartree = 27.211 eV.

### 3.8. Statistical Analysis and Data Reporting

All quantitative experiments were performed in independent triplicates where applicable, and the results are presented as mean ± standard deviation (SD). Error bars in the quantitative figures represent the SD of triplicate measurements. For adsorption kinetics and isotherm analyses, model reliability was evaluated using goodness-of-fit parameters, including R^2^ values, agreement between experimental and calculated adsorption capacities, and the consistency of fitted model parameters.

## 4. Conclusions

The H_2_O_2_ treatment substantially increased the apparent Cr(VI)-derived retention performance of biochar, as indicated by a Langmuir-fitted/extrapolated apparent retention capacity q_m_ of 77.44 mg/g at 328 K. This value is used as a model-derived comparative parameter rather than as a directly measured maximum uptake. The combined kinetic, electrokinetic, FTIR, and XPS results indicate that the higher Cr(VI)-derived retention of H-BC is not attributable to increased electrostatic affinity for chromate oxyanions. Instead, the findings support a reaction-coupled interfacial process involving short-range non-electrostatic interactions, partial Cr(VI) reduction, and stabilization of surface-associated Cr(III) at oxygenated surface domains [5,43]. This interpretation reconciles the enhanced retention performance with the net negative surface charge of H-BC under the experimental conditions.

DFT-assisted electronic-structure analysis provided qualitative support for the enhanced electronic responsiveness of the H-BC fragment model. Compared with BC, H-BC exhibited a lower HOMO–LUMO energy gap, decreasing from 2.6615 to 1.7482 eV, suggesting that H_2_O_2_ functionalization increased the electronic polarizability/reactivity tendency of the representative fragment [36,37]. These calculations were not used as direct evidence for chromate adsorption, charge transfer, or the Cr(VI)-to-Cr(III) reduction pathway. Apparent thermodynamic indicators, including ΔG < 0 and ΔH = 5.58 kJ/mol for H-BC, suggest that elevated temperature favored the overall Cr(VI)-derived retention process under the tested conditions. Because these indicators were derived from fitted K_L_ values that were not converted to dimensionless standard-state constants, they should not be used to separate adsorption, reduction, and mass-transfer contributions [1,28]. Dynamic continuous-flow column leaching and accelerated wet–dry (W–D) aging experiments further showed that H-BC achieved a maximum filtered-Cr(VI)-based retention efficiency of 99.98%, indicating strong suppression of the operationally filtered Cr(VI) fraction under hydraulic flushing and cyclic drying–rewetting stress [2,12]. Evaluations of spatial application strategies indicated that uniform incorporation of H-BC improved soil–biochar contact and was more effective than stratified placement in limiting vertical filtered-Cr(VI) migration [2,39].

Future studies should establish a speciation-resolved chromium balance by measuring Cr(VI), dissolved Cr(III), filtered and unfiltered acid-digested total Cr in solution/leachate, and solid-phase Cr fractions obtained by sequential extraction. Because BC/H-BC would function primarily as an in situ soil amendment rather than a recoverable adsorbent, TCLP-type acetic acid leaching should also be included to assess post-stabilization leachability under field-relevant acidic conditions. In summary, oxidatively functionalized H-BC is a promising biomass-derived material for reducing Cr(VI) mobility in dynamic environmental settings, while further total-Cr, Cr-speciation, and TCLP-based evaluations are needed before field-scale application can be fully assessed.

## Figures and Tables

**Table 1:** N_2_ adsorption–desorption isotherms and pore-size distribution of BC and H-BC.

Sample	BET Surface Area (m^2^/g)	Total Pore Volume (mL/g)	Dominant Pore-Size Peak (nm)	Textural Feature
BC	503.7	0.5674	4.3–4.5	Mesoporous biochar
H-BC	317.1	0.3343	4.3–4.5	Lower-area OFG-enriched mesoporous biochar

**Table 2:** Kinetic parameters and model-selection criteria for Cr(VI) adsorption on BC and H-BC (C_0_ = 106.06 mg/L, T = 25 °C), determined using pseudo-first-order and pseudo-second-order models.

Adsorbent	q_e,exp_ (mg/g)	PFO						PSO					
		q_e,cal1_ (mg/g)	k_1_ (min^−1^)	R_1_^2^	RMSE	AIC	BIC	q_e,cal2_ (mg/g)	k_2_ (g/ (mg∙min))	R_2_^2^	RMSE	AIC	BIC
BC-Cr(VI)	13.3623	12.5915	0.1212	0.9797	0.5659	−7.39	−6.78	13.4346	0.0140	0.9986	0.1504	−33.89	−33.29
H-BC-Cr(VI)	15.2230	14.4409	0.1059	0.9775	0.6900	−3.42	−2.82	15.4665	0.01045	0.9992	0.1284	−37.05	−36.45

q_e,exp_ represents the experimentally measured equilibrium capacity, and q_e,cal_ represents the fitted equilibrium capacity. For the PFO model, k is k_1_ (min^−1^); for the PSO model, k is k_2_ [g/(mg·min)]. RMSE, AIC, and BIC were calculated from the residual sum of squares between experimental and fitted q_t_ values. Lower RMSE, AIC, and BIC values indicate better empirical model performance. The PFO and PSO models are empirical kinetic models and are not used as standalone evidence of chemisorption or electron transfer.

**Table 3:** Parameters for the intra-particle diffusion (IPD) model in the adsorption of Cr(VI).

Adsorbent	Film Diffusion			Intra-Particle Diffusion			Equilibrium		
	K_p1_	C_1_	R^2^	K_p2_	C_2_	R^2^	K_p3_	C_3_	R^2^
BC-Cr(VI)	2.8382	0.0690	0.9929	0.3783	9.2246	0.9983	0.0730	11.8622	0.9977
H-BC-Cr(VI)	3.1070	0.0745	0.9996	0.6666	9.8099	0.9713	0.0644	14.0290	0.8696

The units of k_p1_, k_p2_, and k_p3_: mg · g^−1^ · min^−1/2^.

**Table 4:** Boyd-model fitting parameters for Cr(VI) adsorption kinetics on BC and H-BC.

Adsorbent	Intercept	Slope	RSS	Pearson’s r	R^2^
BC-Cr(VI)	0.75077	0.01416	0.88723	0.96716	0.93539
H-BC-Cr(VI)	0.34730	0.02313	1.17799	0.98324	0.96676

The Boyd plots were obtained by linear regression of B_t_ versus contact time. A non-zero intercept indicates that intra-particle diffusion was not the sole rate-controlling step and that external film diffusion contributed to the overall Cr(VI) uptake process.

**Table 5:** Apparent isotherm fitting parameters and temperature-dependent retention indicators for Cr(VI)-derived retention by BC and H-BC.

Adsorbent	T	Langmuir Model			Freundlich Model			Apparent Thermodynamic Indicators
		q_m_	K_L_	R^2^	K_F_	*n*	R^2^	ΔG
BC-Cr(VI)	25 °C	11.2247	0.0040	0.9975	0.7074	1.2313	0.9393	−13.23
H-BC-Cr(VI)		15.3226	0.0654	0.9939	1.6628	2.2864	0.9865	−20.16
BC-Cr(VI)	40 °C	13.6588	0.1887	0.9980	3.4074	2.7659	0.8948	−23.94
H-BC-Cr(VI)		20.4748	0.1025	0.9993	3.6088	2.3625	0.9632	−22.35
BC-Cr(VI)	55 °C	27.8367	0.1280	0.9957	3.5656	2.5442	0.9912	−24.02
H-BC-Cr(VI)		77.4411	0.0813	0.9997	4.5216	2.0893	0.9982	−22.78

The units of q_m_, K_L_, K_F_ and ΔG are mg/g, L/mg, (mg · g^−1^)/(L · mg^−1^)^1/n^ and kJ/mol. q_m_ represents the Langmuir-derived apparent retention capacity under the assumptions of the model. Because Cr(VI) uptake involved adsorption-coupled partial reduction and no clear saturation plateau was reached, q_m_ should not be interpreted as an experimentally observed maximum capacity or evidence of reversible thermodynamic equilibrium [29,30]. Following Lima et al. [31], the apparent ΔH and ΔS values are not interpreted as rigorous adsorption thermodynamic parameters, as the fitted constants were not treated as dimensionless thermodynamic equilibrium constants and the Cr(VI) removal process involved coupled retention–reduction.

**Table 6:** Comparison of reported Langmuir-type fitted Cr(VI) adsorption/retention capacities of selected biochar-based adsorbents under the reported experimental conditions.

Adsorbents	Adsorption Capacity (mg/g)	Initial Cr(VI) Concentration	Adsorbent Concentration	pH	Temperature	Reference
	Cr (VI)					
Corncob biochar (CCBC)	38.13	25–225 mg/L	2.0 g/L	2.0	34 °C	[32]
Brewery spent grain biochar (BSG AT-5)	31.87	10–160 mg/L	2.0 g/L	2.0	25 °C	[33]
Ball-milled wheat straw biochar (BM-WB)	52.21	10–110 mg/L	2.0 g/L	2.0	45 °C	[34]
H_2_O_2_-modified woody biochar (H-BC)	77.44	17.67–106.06 mg/L	5.0 g/L	6.0 ± 0.1	55 °C	This study

**Table 7:** Frontier molecular orbital energies and HOMO–LUMO gaps of representative BC and H-BC fragment models.

Model	E_HOMO_ (Ha)	E_LUMO_ (Ha)	ΔE (Ha)	ΔE (eV)
BC	−0.173588	−0.075780	0.097808	2.6615
H-BC	−0.160941	−0.096696	0.064250	1.7482

## Data Availability

The data presented in this study are available from the corresponding author upon reasonable request.

## References

[1] Yuan X., Li J., Luo L., Zhong Z., Xie X. (2023). Advances in Sorptive Removal of Hexavalent Chromium (Cr(VI)) in Aqueous Solutions Using Polymeric Materials. Polymers.

[2] Hou R., Wang L., Shen Z., Alessi D.S., Hou D. (2021). Simultaneous Reduction and Immobilization of Cr(VI) in Seasonally Frozen Areas: Remediation Mechanisms and the Role of Ageing. J. Hazard. Mater..

[3] Chen J., Zhou J., Zheng W., Leng S., Ai Z., Zhang W., Yang Z., Yang J., Xu Z., Cao J. (2024). A Complete Review on the Oxygen-Containing Functional Groups of Biochar: Formation Mechanisms, Detection Methods, Engineering, and Applications. Sci. Total Environ..

[4] Yang J., Song Y., Yue Y., Liu W., Che Q., Chen H., Ma H. (2021). Chemically Dual-Modified Biochar for the Effective Removal of Cr(VI) in Solution. Polymers.

[5] Yoon S., Bae S. (2025). Mechanistic Study of Cr(VI) Reduction by Functionalized Biochar: Distinct Roles of Persistent Free Radicals, Ascorbate Moieties, and Oxygen Functional Groups. Chem. Eng. J..

[6] Chen N., Cao S., Zhang L., Peng X., Wang X., Ai Z., Zhang L. (2021). Structural Dependent Cr(VI) Adsorption and Reduction of Biochar: Hydrochar versus Pyrochar. Sci. Total Environ..

[7] Wen J., Xue Z., Yin X., Wang X. (2022). Insights into Aqueous Reduction of Cr(VI) by Biochar and Its Iron-Modified Counterpart in the Presence of Organic Acids. Chemosphere.

[8] Qi G., Pan Z., Zhang X., Wang H., Chang S., Wang B., Gao B. (2024). Novel Pretreatment with Hydrogen Peroxide Enhanced Microwave Biochar for Heavy Metals Adsorption: Characterization and Adsorption Performance. Chemosphere.

[9] Yang C., Wu H., Cai M., Zhou Y., Guo C., Han Y., Zhang L. (2023). Valorization of Biomass-Derived Polymers to Functional Biochar Materials for Supercapacitor Applications via Pyrolysis: Advances and Perspectives. Polymers.

[10] Zheng C., Yang Z., Si M., Zhu F., Yang W., Zhao F., Shi Y. (2021). Application of Biochars in the Remediation of Chromium Contamination: Fabrication, Mechanisms, and Interfering Species. J. Hazard. Mater..

[11] Zhang X., He Y., Li Q., Liao Q., Si M., Yang Z., Yang W. (2024). Simulated Scenario Models to Assess the Long-Term Effects of Cr(VI)-Contaminated Soils Remediated with Typical Iron-Bearing Reductants. J. Soils Sediments.

[12] Chen Z., Chen Y., Liang J., Sun Z., Zhao H., Huang Y. (2024). The Release and Migration of Cr in the Soil under Alternating Wet–Dry Conditions. Toxics.

[13] Ilić M., Haegel F.-H., Lolić A., Nedić Z., Tosti T., Ignjatović I.S., Linden A., Jablonowski N.D., Hartmann H. (2022). Surface Functional Groups and Degree of Carbonization of Selected Chars from Different Processes and Feedstock. PLoS ONE.

[14] Liepins K., Volperts A., Dobele G., Plavniece A., Bikovens O., Sansonetti E., Zhurinsh A. (2024). Enhancing the Wetting Properties of Activated Biochar by Oxidation with Hydrogen Peroxide. Chemistry.

[15] Zhang J., Amonette J.E., Flury M. (2021). Effect of Biochar and Biochar Particle Size on Plant-Available Water of Sand, Silt Loam, and Clay Soil. Soil Tillage Res..

[16] Greczynski G., Hultman L. (2021). The Same Chemical State of Carbon Gives Rise to Two Peaks in X-Ray Photoelectron Spectroscopy. Sci. Rep..

[17] Fu W., Wu M., Chen Q., Liang Y., Peng H., Zeng L., Pan B. (2024). The Role of Superoxide Anion to Cr(VI) Reduction by Pine Biochar. J. Hazard. Mater..

[18] Yang Z., Wang J., Zhao N., Pang R., Zhao C., Deng Y., Yang D., Jiang H., Wu Z., Qiu R. (2024). A Novel Biochar-Based 3D Composite for Ultrafast and Selective Cr(VI) Removal in Electroplating Wastewater. Biochar.

[19] Ren J., Huang H., Zhang Z., Xu X., Zhao L., Qiu H., Cao X. (2023). Enhanced Microbial Reduction of Cr(VI) in Soil with Biochar Acting as an Electron Shuttle: Crucial Role of Redox-Active Moieties. Chemosphere.

[20] Qiu Y., Zhang Q., Gao B., Li M., Fan Z., Sang W., Hao H., Wei X. (2020). Removal Mechanisms of Cr(VI) and Cr(III) by Biochar Supported Nanosized Zero-Valent Iron: Synergy of Adsorption, Reduction and Transformation. Environ. Pollut..

[21] Kosmulski M. (2023). The pH Dependent Surface Charging and Points of Zero Charge. X. Update. Adv. Colloid Interface Sci..

[22] Chen D., Arancibia-Miranda N., Escudey M., Fu J., Lu Q., Amon C.H., Galatro D., Guzmán A.M. (2023). Nonlinear Dependence (on Ionic Strength, pH) of Surface Charge Density and Zeta Potential in Microchannel Electrokinetic Flow. Heliyon.

[23] Frangenberg M., Schmidt A.M., Wilkens J. (2025). New Experimental Approach for the Proper Consideration of Stagnant and Diffuse Layer Conductivity in the Zeta Potential Determination. Langmuir.

[24] Qu J., Wang Y., Tian X., Jiang Z., Deng F., Tao Y., Jiang Q., Wang L., Zhang Y. (2021). KOH-Activated Porous Biochar with High Specific Surface Area for Adsorptive Removal of Chromium (VI) and Naphthalene from Water: Affecting Factors, Mechanisms and Reusability Exploration. J. Hazard. Mater..

[25] Qu J., Yang Q., Gong W., Li M., Cao B. (2022). Simultaneous Removal of Cr(VI) and Phenol from Water Using Silica-Di-Block Polymer Hybrids: Adsorption Kinetics and Thermodynamics. Polymers.

[26] Xiao X., Zhou F., Yang C., Lu L., Zhao Y., Tu B., Li L. (2025). Adsorption and Reduction of Cr(VI) by C-P-O Groups in Biochar: Performance and Mechanisms. Solid State Sci..

[27] Yan L., Dong F.-X., Lin X., Zhou X.-H., Kong L.-J., Chu W., Diao Z.-H. (2021). Insights into the Removal of Cr(VI) by a Biochar–Iron Composite from Aqueous Solution: Reactivity, Kinetics and Mechanism. Environ. Technol. Innov..

[28] Masuku M., Nure J.F., Atagana H.I., Hlongwa N., Nkambule T.T.I. (2024). Pinecone Biochar for the Adsorption of Chromium (VI) from Wastewater: Kinetics, Thermodynamics, and Adsorbent Regeneration. Environ. Res..

[29] Li Y., Li Q., Wu C., Luo X., Yu X., Chen M. (2020). The Inappropriate Application of the Regression Langmuir Qm for Adsorption Capacity Comparison. Sci. Total Environ..

[30] Hu Q., Lan R., He L., Liu H., Pei X. (2023). A Critical Review of Adsorption Isotherm Models for Aqueous Contaminants: Curve Characteristics, Site Energy Distribution and Common Controversies. J. Environ. Manag..

[31] Lima E.C., Hosseini-Bandegharaei A., Moreno-Piraján J.C., Anastopoulos I. (2019). A Critical Review of the Estimation of the Thermodynamic Parameters on Adsorption Equilibria. Wrong Use of Equilibrium Constant in the van’t Hoof Equation for Calculation of Thermodynamic Parameters of Adsorption. J. Mol. Liq..

[32] Nguyen D.-K., Ly-Tran Q.-B., Dinh V.-P., Duong B.-N., Nguyen T.-P.-T., Nguyen Kim Tuyen P. (2024). Adsorption Mechanism of Aqueous Cr(VI) by Vietnamese Corncob Biochar: A Spectroscopic Study. RSC Adv..

[33] Zewde Z., Asere T.G., Yitbarek M. (2024). Porous Biochars Derived from Brewery Waste for the Treatment of Cr(VI)-Contaminated Water. PLoS ONE.

[34] Tan Y., Wang J., Zhan L., Yang H., Gong Y. (2024). Removal of Cr(VI) from Aqueous Solution Using Ball Mill Modified Biochar: Multivariate Modeling, Optimization and Experimental Study. Sci. Rep..

[35] Tran H.N. (2022). Improper Estimation of Thermodynamic Parameters in Adsorption Studies with Distribution Coefficient *K*_D_ (*q*_e_/*C*_e_) or Freundlich Constant (*K*_F_): Considerations from the Derivation of Dimensionless Thermodynamic Equilibrium Constant and Suggestions. Adsorpt. Sci. Technol..

[36] He D., Luo Y., Zhu B. (2024). Feedstock and Pyrolysis Temperature Influence Biochar Properties and Its Interactions with Soil Substances: Insights from a DFT Calculation. Sci. Total Environ..

[37] He D., Liu X., Hu D., Lei P., Zhang J., Dong Z., Zhu B. (2025). Density Functional Theory Calculation for Understanding the Roles of Biochar in Immobilizing Exchangeable Al^3+^ and Enhancing Soil Quality in Acidic Soils. Ecotoxicol. Environ. Saf..

[38] Chen M., Chen X., Xu X., Xu Z., Zhang Y., Song B., Tsang D.C.W., Xu N., Cao X. (2022). Biochar Colloids Facilitate Transport and Transformation of Cr(VI) in Soil: Active Site Competition Coupling with Reduction Reaction. J. Hazard. Mater..

[39] Zhou Z., Liu P., Wang S., Finfrock Y.Z., Ye Z., Feng Y., Li X. (2022). Iron-Modified Biochar-Based Bilayer Permeable Reactive Barrier for Cr(VI) Removal. J. Hazard. Mater..

[40] Wang L., Luo Y., Pang J., Li Y., Wu H., Jiang X., Tong J., Shi J. (2023). Fe-Biochar for Simultaneous Stabilization of Chromium and Arsenic in Soil: Rational Design and Long-Term Performance. Sci. Total Environ..

[41] Xu Z., Yu Y., Xu X., Tsang D.C.W., Yao C., Fan J., Zhao L., Qiu H., Cao X. (2022). Direct and Indirect Electron Transfer Routes of Chromium(VI) Reduction with Different Crystalline Ferric Oxyhydroxides in the Presence of Pyrogenic Carbon. Environ. Sci. Technol..

[42] Liu T., Wei R., Li J., Xie W., Sun S., Deng T., Wang S., Tang Y., Lin Q., Ni Z. (2024). Fe (Hydr)Oxides and Organic Colloids Mediate Colloid-Bound Chromium Mobilization in Cr(VI) Contaminated Paddy Soil. Environ. Pollut..

[43] Ekere I., Johnston B., Tchuenbou-Magaia F., Townrow D., Wojciechowski S., Marek A., Zawadiak J., Duale K., Zieba M., Sikorska W. (2022). Bioconversion Process of Polyethylene from Waste Tetra Pak^®^ Packaging to Polyhydroxyalkanoates. Polymers.

[44] Wei Y., Yuan C., Xu X., Chen X., Ren Z., Gui X., Zhao L., Qiu H., Cao X. (2022). Colloid Formation and Facilitated Chromium Transport in the Coastal Area Soil Induced by Freshwater and Seawater Alternating Fluctuations. Water Res..

[45] Eckbo C., Okkenhaug G., Hale S.E. (2022). The Effects of Soil Organic Matter on Leaching of Hexavalent Chromium from Concrete Waste: Batch and Column Experiments. J. Environ. Manag..

[46] Shi J., McGill W.B., Chen N., Rutherford P.M., Whitcombe T.W., Zhang W. (2020). Formation and Immobilization of Cr(VI) Species in Long-Term Tannery Waste Contaminated Soils. Environ. Sci. Technol..

[47] Yang J., Guo Q., Li L., Wang R., Chen Y., Wang X. (2023). Insights into the Evolution of Cr(VI) Species in Long-Term Hexavalent Chromium Contaminated Soil. Sci. Total Environ..

[48] Hu S., Li D., Man Y., Wen Y., Huang C. (2021). Evaluation of Remediation of Cr(VI)-Contaminated Soils by Calcium Polysulfide: Long-Term Stabilization and Mechanism Studies. Sci. Total Environ..

[49] Xie L., Chen Q., Liu Y., Ma Q., Zhang J., Tang C., Duan G., Lin A., Zhang T., Li S. (2023). Enhanced Remediation of Cr(VI)-Contaminated Soil by Modified Zero-Valent Iron with Oxalic Acid on Biochar. Sci. Total Environ..

[50] Zhang L., He F., Guan Y. (2022). Immobilization of Hexavalent Chromium in Contaminated Soil by Nano-Sized Layered Double Hydroxide Intercalated with Diethyldithiocarbamate: Fraction Distribution, Plant Growth, and Microbial Evolution. J. Hazard. Mater..

[51] Yang Z., Zhang X., Jiang Z., Li Q., Huang P., Zheng C., Liao Q., Yang W. (2021). Reductive Materials for Remediation of Hexavalent Chromium Contaminated Soil—A Review. Sci. Total Environ..

[52] Park J.H. (2020). Contrasting Effects of Cr(III) and Cr(VI) on Lettuce Grown in Hydroponics and Soil: Chromium and Manganese Speciation. Environ. Pollut..

[53] Yang Y., Peng Y., Ma Y., Chen G., Li F., Liu T. (2022). Effects of Aging and Reduction Processes on Cr Toxicity to Wheat Root Elongation in Cr(VI) Spiked Soils. Environ. Pollut..

[54] Ao M., Chen X., Deng T., Sun S., Tang Y., Morel J.L., Qiu R., Wang S. (2022). Chromium Biogeochemical Behaviour in Soil-Plant Systems and Remediation Strategies: A Critical Review. J. Hazard. Mater..

[55] Ceballos E., Cama J., Soler J.M., Frei R. (2023). Release and Mobility of Hexavalent Chromium in Contaminated Soil with Chemical Factory Waste: Experiments, Cr Isotope Analysis and Reactive Transport Modeling. J. Hazard. Mater..

[56] Gu Y., Chen X., Liu L., Wang S., Yu X., Jia Z., Zhou X. (2023). Cr(VI)-Bioremediation Mechanism of a Novel Strain Bacillus Paramycoides Cr6 with the Powerful Ability to Remove Cr(VI) from Contaminated Water. J. Hazard. Mater..

[57] Tomczyk A., Sokołowska Z., Boguta P. (2020). Biochar Physicochemical Properties: Pyrolysis Temperature and Feedstock Kind Effects. Rev. Environ. Sci. Biotechnol..

[58] Tomczyk A., Kondracki B., Szewczuk-Karpisz K. (2023). Chemical Modification of Biochars as a Method to Improve Its Surface Properties and Efficiency in Removing Xenobiotics from Aqueous Media. Chemosphere.

[59] Mutabazi E., Qiu X., Song Y., Li C., Jia X., Hakizimana I., Niu J., Nuramkhaan M., Zhao Y. (2024). Cr(VI) Adsorption on Activated Carbon, Sludge Derived Biochar, and Peanut Shells Derived Biochar: Performance, Mechanisms during the Reuse Process and Site Energy Distribution Analysis. J. Water Process Eng..

[60] Dikobe J., Melato F.A., Adlem C.J.L., Netshiongolwe K. (2024). Determination of Chromium Species in Water Using Diphenylcarbazide with a Sequential Spectrophotometric Discrete Robotic Analyser. Heliyon.

[61] Singh S., Anil A.G., Naik T.S.S.K., U. B., Khasnabis S., Nath B., Kumar V., Subramanian S., Singh J., Ramamurthy P.C. (2022). Mechanism and Kinetics of Cr(VI) Adsorption on Biochar Derived from Citrobacter Freundii under Different Pyrolysis Temperatures. J. Water Process Eng..

[62] Murphy O.P., Vashishtha M., Palanisamy P., Kumar K.V. (2023). A Review on the Adsorption Isotherms and Design Calculations for the Optimization of Adsorbent Mass and Contact Time. ACS Omega.

[63] Agarwal S., Wadhwa S., Singhal S., Bhogal A. (2026). Waste-to-Resource Strategy: MnOx—Modified Pine Biochar for Cr(VI) Ions Removal from Water. Mater. Sci. Eng. B.

[64] Nguyen D.-K., Nguyen Q.-T., Dinh V.-P. (2025). Integrating Artificial Intelligence with Kinetic Studies for Cr(VI) Removal Using Young Durian Fruit Biochar: A Random Forest Regressor Approach. RSC Adv..

[65] Wang B., Ci S., Zhou M., Di C., Yu J., Zhu B., Qiao K. (2023). Effects of Hygrothermal and Salt Mist Ageing on the Properties of Epoxy Resins and Their Composites. Polymers.

